# Comparative genomics reveals common diversity and adaptation to harsh environments in the Arabian Peninsula indigenous chickens

**DOI:** 10.1111/age.70014

**Published:** 2025-05-02

**Authors:** Abdulwahad Assiri, Adriana Vallejo‐Trujillo, Mohammed Al‐Abri, Hussain Bahbahani, Faisal Almathen, Abulgasim Ahbara, Waleed Al Marzooqi, Abdulfatai Tijjani, Raman Lawal, Olivier Hanotte

**Affiliations:** ^1^ Department of Livestock and Fish Production King Faisal University Al‐Ahsa Saudi Arabia; ^2^ School of Life Sciences University of Nottingham Nottingham UK; ^3^ Centre for Tropical Livestock Genetics and Health (CTLGH), The Roslin Institute The University of Edinburgh Edinburgh UK; ^4^ Department of Animal and Veterinary Sciences Sultan Qaboos University Muscat Oman; ^5^ Department of Biological Sciences, Faculty of Sciences, Sh. Sabah Al‐Salem campus Kuwait University Al‐Shadadiya Kuwait; ^6^ Department of Veterinary Public Health and Animal Husbandry, College of Veterinary Medicine King Faisal University Al‐Ahsa Saudi Arabia; ^7^ Camel Research Centre King Faisal University Al‐Ahsa Saudi Arabia; ^8^ Animal and Veterinary Sciences Scotland's Rural College (SRUC) Midlothian UK; ^9^ Department of Zoology, Faculty of Sciences Misurata University Misurata Libya; ^10^ The Feinstein Institutes of Medical Research (Northwell Health) Manhasset New York USA; ^11^ The Jackson Laboratory Bar Harbor Maine USA; ^12^ Centre for Tropical Livestock Genetics and Health (CTLGH) ILRI Addis Ababa Ethiopia

**Keywords:** Arabian Peninsula indigenous chicken, diversity, environmental adaptation, signature of selection

## Abstract

Identifying genomic regions under selection is crucial for comprehending the evolutionary history of the domestic chicken. Arabian Peninsula (AP) indigenous chickens are mostly found outdoors, being reared alongside other livestock for production purposes. These birds show high resilience to extreme temperatures (hot and cold), typical of the desert environment. The selection pressures responsible for unique local adaptations in these birds remain largely unidentified. Here, we aimed to investigate the genome diversity and structure of 15 indigenous chicken populations including 13 populations from the AP (*n* = 5), Ethiopia (*n* = 6), and the People's Republic of China (*n* = 2). We also included two commercial chicken populations, Fayoumi (selected for heat tolerance) and Chantecler (known for its cold tolerance). Principal component (PC) analysis separated all the populations based on their geographic areas of origin. PC1 separates the Ethiopian populations from the Chinese and AP populations, while PC2 separates the AP populations from the Chantecler, and the Ethiopian populations from the Dulong and Chantecler. The genome‐wide signatures of analyses identified many candidate regions under positive selection. They include genes that may be associated with thermotolerance. These are involved in energy balance and metabolism (*SUGCT*, *HECW1*, *MMADHC*), cells apoptosis (*APP*, *SRBD1*, *NTN1*, *PUF60*, *SLC26A8*, *DAP*, *SUGCT*), angiogenesis (*RYR2*, *LDB2*, *SOX5*), skin protection to solar radiation (*FZD10*, *BCO2*, *WNT5B*, *COL6A2*, *SIRT1*) as well as growth (*NELL1*). Our findings suggest that Arabian chicken populations have a distinct gene pool polymorphism in relation to their adaptation to the harsh climatic environments of the AP.

## INTRODUCTION

Indigenous chicken populations, shaped over millennia through adaptation, offer a fascinating insight into the genomic signatures of natural selection in response to varying thermal environments. These adaptable birds have developed unique traits that enable them to survive and reproduce in diverse climates, highlighting the intricate interplay between genetic evolution and environmental pressures (Lawler, 2016). For example, Perini et al. (2020) review the molecular pathways related to heat stress, while Juiputta et al. (2023) discuss the genetic strategies to improve heat tolerance and sustain productivity in tropical poultry farming. Moreover, Nawaz et al. (2024) assess crucial genes and pathways linked to heat tolerance, such as the heat shock response, antioxidant defense systems, immune function, and cellular homeostasis.

They thrive in hot climates, such as the arid regions of sub‐Saharan Africa or the scorching deserts of the Arabian Peninsula (AP), having developed a suite of adaptations to endure the relentless heat stress (Fathi, Al‐Homidan, Abou‐Emera & Al‐Moshawah, 2017; Kanyama et al., 2022). These adaptations encompass various physiological, behavioral, and genetic polymorphisms aimed at maintaining homeostasis in high‐temperature environments (Cheng & Muir, 2005). Notable among these are efficient thermoregulatory mechanisms, such as increased feather reflectance and the capacity to dissipate heat through behaviors such as shade‐seeking and panting (Soleimani et al., 2011).

Genomic studies have revealed genetic signatures associated with heat tolerance, emphasizing substantial variations in the genes responsible for adapting to heat stress among indigenous chicken populations. For example, Guo et al. (2022) identified several selective sweeps harboring genes under selection (*FABP2*, *RAMP3*, *SUGCT*, and *TSHR*) in indigenous chickens from tropical regions (northern China, Indonesia, Sri Lanka, and Thailand), which may be associated with adaptation to higher ambient temperatures. They specifically found a missense mutation in *TSHR* which could enhance the heat tolerance in chicken. Zhuang et al. (2020) identified genes associated with thermotolerance in Taiwan indigenous chickens, including genes involved in DNA repair, cellular stress responses, apoptosis, and metabolic oxidative stress. Lawal et al. (2018) observed that the *KCNMA1* gene is within a selection signature region in Saudi Arabian and Sri Lankan chicken populations. This gene is associated with the response to hypoxia challenge and the regulation of smooth muscle contraction. Several genes involved in the adaptation to climate were also identified in Chinese native chickens including *APP*, *FABP1*, *SMYD1*, *UBE4B*, *NALCN*, *PDGFRA*, *NRP1*, *CORIN*, CLPTM*1L*, *CRADD*, *PARK2*, *SIM2*, *AHR*, *ESRRG*, *IL18*, and *BVES* (Gu et al., 2020). In Nigerian indigenous chicken, genes that may be linked to heat stress adaptation (e.g., *ILF3*, *HSF1*, *SLC44A2*, *SFTPB*, *HIF3A*, *CDC37*, and *TSHR*) were recently reported (Rachman et al., 2024).

Moreover, genomic investigations have pinpointed genetic variants associated with cold adaptation, including variants governing feather development, lipid metabolism, and cold‐induced thermogenesis (Wang et al., 2015). These adaptations are indispensable for surviving in cold climates, where energy conservation and heat retention are critical. In a comprehensive genome‐wide analysis by Xu et al. (2021), two candidate selected regions encompass genes that might be linked to adaptation to cold stress in Chantecler were identified. These regions include the *ME3* gene, involved in fat metabolism, and *ZNF536*, which is linked to the nervous system.

The AP climatic circumstances offer an exceptional opportunity to unravel the genetic determinants that drive adaptations to challenging environmental conditions. The AP exhibits a diverse range of seasonal and regional climate patterns. Specifically, certain regions suffer scorching summer temperatures that exceed 50°C (122°F), resulting in an exceedingly hot and arid environment (Abou‐Shaara et al., 2013). Conversely, temperatures may plummet to freezing during winter, especially in the northern areas. Remarkably, despite the formidable environmental challenges they face, indigenous chickens not only survive but also coexist harmoniously with humans in these harsh conditions (Fathi, Al‐Homidan, Motawei, et al., 2017; Soliman et al., 2016). With no comprehensive statistical sources on their population size or the number of farms raising them, the fate of indigenous chicken genetic resources in the AP remains uncertain. Fathi, Al‐Homidan, Motawei, et al. (2017) highlighted that these native breeds are at risk of endangerment due to genetic erosion caused by uncontrolled crossbreeding with exotic breeds, further worsened by inadequate management practices.

Here, we investigate indigenous chickens from the AP aiming to bridge the knowledge gap in chicken genomic diversity and adaptations in the region. While there is observational evidence that indigenous chicken populations are well‐adapted to their challenging environments, the genetic mechanisms driving these adaptations remain understudied (Muchadeyi & Dzomba, 2017). Additionally, minimal effort has been made to dissect the genetic diversity and make‐up of indigenous chickens in the AP region.

## MATERIALS AND METHODS

### Sampling and sequencing

Genome sequences of 156 samples from 15 indigenous chicken populations from various geographic regions that span different climate zones (Table 1 and Table S1) were included in the study. Ten of these populations have been previously studied, while five populations are new data. These include Fayoumi (*n* = 10), originally from Egypt, Black feather (BL‐KFU; *n* = 13), and Brown feather (BR‐KFU; *n* = 15), raised at the King Faisal University Research and Training Station in the eastern region of the Kingdom of Saudi Arabia. The Fayoumi birds included eight females and two males, randomly selected from a flock of 700 birds. BL‐KFU birds and the BR‐KFU birds included 10 females and three males each, randomly selected from a flock of 500 birds and 1000 birds, respectively. Also, we sampled indigenous birds at the farm level for two indigenous populations. These birds were Buqyiq (BU‐VI; *n* = 13) from Buqyiq city in Saudi Arabia and Omani birds (*n* = 20) from the Barka State in Oman. BU‐VI included 10 females and three males. They were sampled from a single farm with an indigenous chicken population size of 100 birds. All Omani chicken were females. Genome data from the remaining samples were downloaded from public genome databases (Table S1). Geographic positioning system coordinates were recorded for the new populations (see Table S1). For the newly sequenced genomes, blood samples were collected from the wing vein in EDTA tubes. The DNA was extracted using Qiagene DNeasy Blood and Tissue Kits following the manufacturer's protocol (https://www.qiagen.com/ca/resources/download.aspx?id=63e22fd7‐6eed‐4bcb‐8097‐7ec77bcd4de6&lang=en) and resuspended at a final concentration of 50 ng/μl. The DNA was sequenced at NEOGEN (https://www.neogen.com) in the UK for whole‐genome sequencing at 20× coverage on a HiSeq Illumina platform.

**TABLE 1:** Agro‐climatic and geographic origin of the chicken populations included in the study. See Table S1 for further information.

Populations	Sample size	Temperatures range at sampling locations year 2020–2021 https://www.worldclim.org/		Elevation (m)	Average rainfall (mm)	Ecological classification (https://weatherandclimate.com/countries)	Geographic regions (countries)	References	Project access number ENA
		Min (°C)	Max (°C)						
*Hot climate populations*									
Omani	20	19	42	1200	80–100	Subtropical desert/arid	Oman—Barka State	New sequences	PRJEB77573
Saudi	5	17	43	154	74	Subtropical desert/arid	Saudi Arabia	Lawal et al. (2018)	PRJNA453469
Fayoumi	10						Egypt population samples at Alhufuf (Saudi Arabia)	New sequences	PRJEB77573
BR‐KFU	15						Alhufuf (Saudi Arabia)		
BL‐KFU	13								
BU‐VI	12			90					
*Cold climate populations*									
Chantecler	9	−25	20	1652	>1000	Cold/sub‐humid	Quebec (Canada)	Xu et al. (2021)	PRJNA720223
Dulong	10	−6	16	3000	>3000	Tropic high‐altitude cold	Alpine Canyon Belt of the North–South Hengduan mountains (People's Republic of China)	Wang et al. (2020)	PRJNA559932
Tibet	5	−7	23	3650	–	Cold/dry	Tibetan Plateau (People's Republic of China)	Zhang et al. (2016)	PRJNA309581
*Ethiopian populations in cold area*									
Alfa Midir	9	1	19	3451	>1000	Tropic cold/sub‐humid	Central Amhara Mountains (Ethiopia)	Gheyas et al. (2022)	PRJEB39275
Gafera	10	5	25	2592	900–1000				
Negasi Amba	10	3	20	3071	900–1000				
*Ethiopian populations in warm area*									
Gesses	10	11	33	1546	>900	Tropic warm/semi‐arid	Western Amhara Valleys (Ethiopia)	Gheyas et al. (2022)	PRJEB39275
Hugub	9	15	37	979	>900		Eastern Afar (Ethiopia)		
Kido	9	11	33	1418	>900	Tropic warm/arid	Western Amhara Valleys (Ethiopia)		

### Mapping and calling variants

The whole‐genome sequence reads were mapped to the reference genome assembly “GRCg6a”, using the *bwa‐mem* algorithm of the burrows‐wheeler aligner version 0.7.17 (Li & Durbin, 2009). The mapped reads were sorted by coordinates and PCR‐duplicates were marked and removed using the *SortSam* and *MarkDuplicates* options of picard version 2.18.29 (https://broadinstitute.github.io/picard/), respectively. Each sample was then subjected to a base quality score recalibration using dbSNP “known sites” (http://ftp.ensembl.org/pub/release‐104/variation/vcf/gallus_gallus/). This process was aimed at removing errors or misalignments during sequencing and identifying known variants. Single nucleotide polymorphisms (SNPs) were called using the *HaplotypeCaller* algorithim of genome analysis toolkit (gatk) version 4.2.2.0 on GVCF mode (McKenna et al., 2010) in each sample. A workplace for each chromosome was applied using *GenomicsDBImport* algorithm. Joint genotyping was performed to identify variants in all samples simultaneously followed by hard filtering using *VariantFiltration* algorithm for high‐quality SNP calling. Subsequently, bi‐allelic autosomal variants that passed the variant filtration step were selected for the downstream analyses.

### Genomic diversity and population structure

Several methods were used to explore genetic diversity and population structure. vcftools version 0.1.14 (Danecek et al., 2011) was used for the genetic diversity estimations at the genome level, including pairwise population differentiation (*F*_ST_) and heterozygosity. The average genome *F*_ST_ was calculated using 20‐kb windows with a sliding step of 10 kb. Quality control analysis was performed on the called autosomal SNPs for the purpose of genetic diversity and population structure analyses using plink 1.9 (Chang et al., 2015). SNPs with minor allele frequency≤0.05 (4 289 890 SNPs), and genotyping call rate ≤95% were excluded (4143 SNPs). For the genetic diversity analyses, SNPs in pairs with high linkage disequilibrium (squared correlation coefficient *r* ^2^ > 0.1) were further excluded (17 237 390 SNPs). Principal component analysis was performed with remaining 3 374 709 SNPs using *–pca* option in plink 1.9 and plotted using r tidyverse package (Wickham et al., 2019). admixture version 1.3.0 program (Alexander et al., 2009), for up to 12 inferred ancestral clusters (*K*), was used to assess the optimal genome ancestry proportions in the analyzed chicken populations.

### Signatures of selection analyses

We performed across all populations four tests to detect candidate signatures of positive selection including pooled heterozygosity *Hp* (Rubin et al., 2010), integrated haplotype score *iHS* (Voight et al., 2006), population differentiation *F*_ST_ (Akey et al., 2002), and cross‐population extended haplotype homozygosity *XP‐EHH* (Sabeti et al., 2007). We used 20‐kb sliding windows with a 10‐kb step in all methods with a minimum of 20 SNPs per window. The *Hp* and *F*_ST_ values were standardized (*ZHp* and *ZF*_ST_) to ensure consistency across all analyses. *iHS* and *XP‐EHH* analyses were conducted using the HAPBIN software (Ahbara et al., 2022; Maclean et al., 2015) after removing SNPs with missing genotypes. *iHS* and *XP‐EHH* analyses were performed for individual SNPs, and then the mean values were calculated within windows for the standardized *iHS* (*iHS*_std) and *XP‐EHH* (*XP‐EHH*_std) metrics.

### Gene identification and functional annotation

We used the reference genome assembly “GRCg6a”, dbSNP database release 110, (2023), and the Ensembl's “VEP” (McLaren et al., 2016) for SNPs positions and genes identification. The *cut‐off* threshold for outlier detection involved selecting the extreme low 0.001 percentile of *ZHp*, and the extreme high 0.001 percentile for *iHS*, *ZF*_ST_, and *XP‐EHH* values, of their respective empirical distributions.

The identified regions were then consolidated using bedtools v.2.25.0. Genes overlapping with candidate regions were identified based on the 108 Ensembl Genes database using the online Ensembl BioMart tool (http://www.ensembl.org/biomart). Two separate lists were generated: (i) genes overlapping or within signatures of selection regions using within‐population *ZHp* and *iHS* analysis; and (ii) genes overlapping or within signatures of selection identified by between‐population *F*_ST_ and *XP‐EHH* pairwise comparisons. We employed the Database for Annotation, Visualization, and Integrated Discovery (DAVID) software version 6.8 (https://david.ncifcrf.gov/summary.jsp) to scrutinize the gene lists for significant enrichment of genes associated with specific functional categories. DAVID's analysis encompasses various annotation categories, such as Gene Ontology (GO), Biological Process, and GO Molecular Function, to identify enriched biological processes and functions within the gene lists. A significance threshold of an adjusted Benjamini‐corrected *p*‐value of 0.05 was used to determine statistical enrichment. Furthermore, we delved into the biological roles of each annotated gene through an extensive literature search incorporating information from diverse species.

## RESULTS

### Sequencing and variant analysis

Genome sequences of chicken samples from Saudi Arabia, Oman, and Egypt yielded an average of 221–480 million paired sequence reads. The average genome coverage for the sequences ranged from 16.98× to 57.46× (see Table S2). The reads were aligned to the chicken reference genome (GRCg6a), and a joint analysis of all samples was subsequently conducted. This analysis uncovered approximately 25 million high‐quality SNPs for the 15 populations. Combining all populations found that 38.25% of the entire set of SNPs (9.5 million out of 24.9 million) were novel according to dbSNP release 110 (2023). Among the populations examined, Chantecler had the lowest percentage (12.4%) of novel SNPs, and the AP populations BL‐KFU, BR‐KFU, and BU.VI has the highest ones 22.31%, 24.44%, and 24.73%, respectively (Table S3). The observed SNP density across chromosomes is illustrated in Table S4. The average density of SNPs across the autosomes is 26.49 SNPs/kb. Chromosome 22 exhibits the lowest SNP density, while chromosome 31 displays the highest density, closely followed by chromosome 33.

### Population structure and differentiation

**FIGURE 1:**
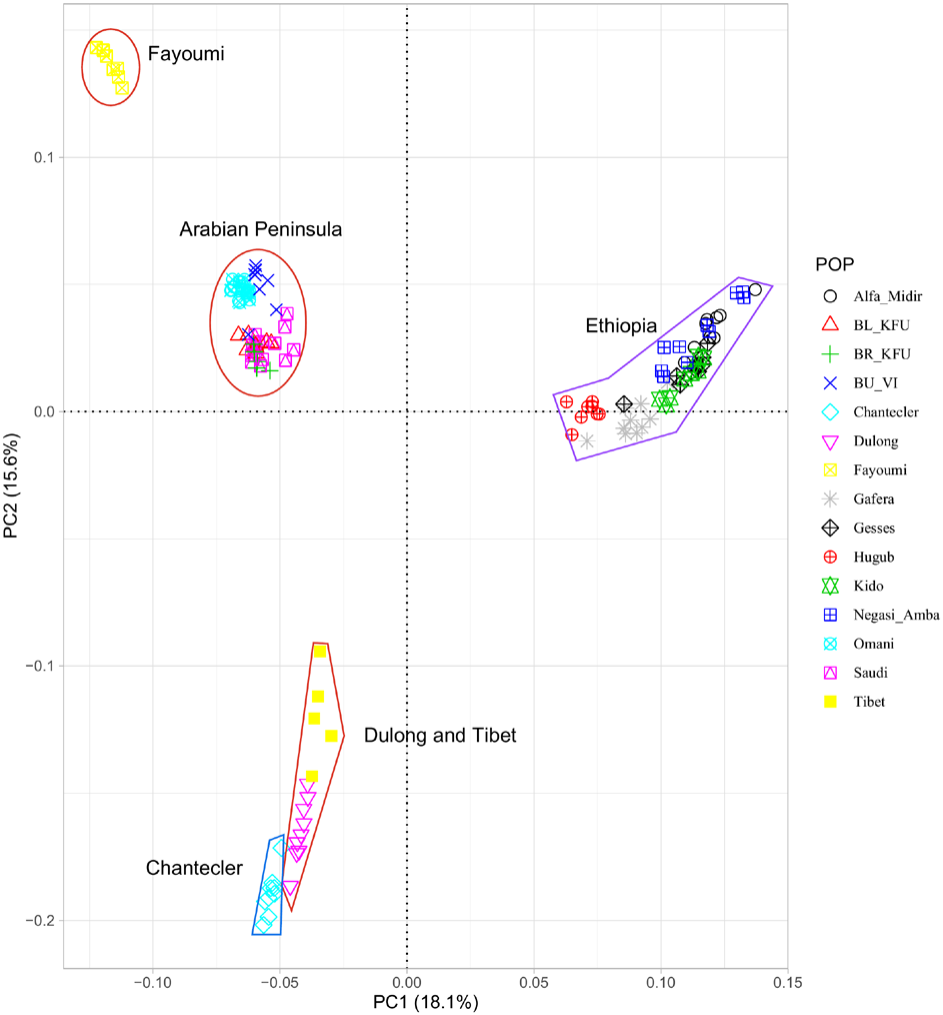
Principal component analyses PC1 vs. PC2 for the studied populations.

**FIGURE 2:**
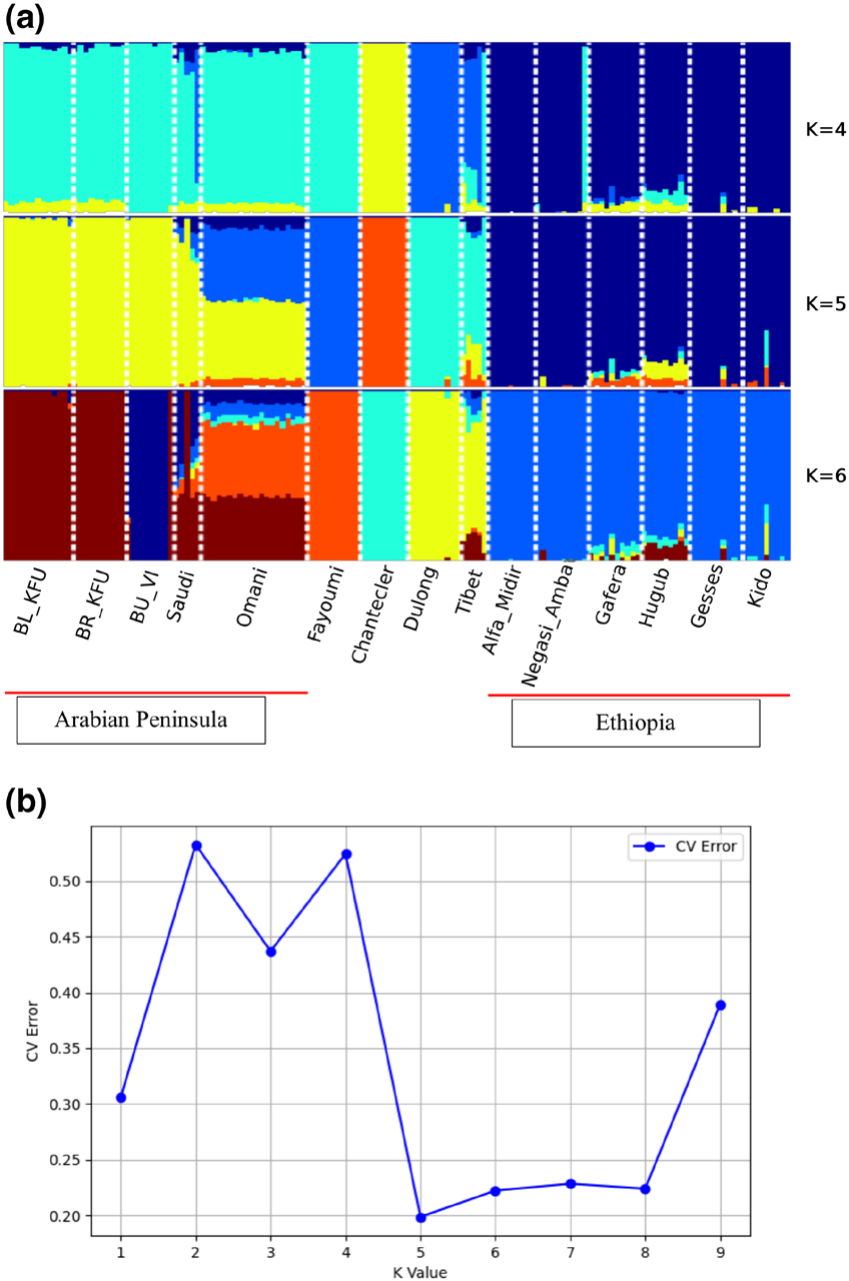
(a) Admixture analyses of the 15 populations (*K* = 4–6). Populations are demarcated with dashed lines. (b) The cross‐validation (CV) error of different K values showing the lowest cross‐validation error (0.199) at *K* = 5.

Population structure was examined using principal component analysis, admixture and genetic differentiation (*F*_ST_) analysis. Principal component (PC)1, accounting for 18.1% of the variation, clearly distinguishes all Ethiopian populations from the remaining populations, while PC2, explaining 15.6% of the total variation, separates the AP and Fayoumi populations from the Chantecler, as well as most Ethiopian populations from the Dulong and Chantecler (Figure 1). The Omani and Saudi Arabian populations lie in an intermediate position in Figure 1. Admixture analysis (*K* = 5) supports a single ancestral gene pool for the Saudi Arabian populations, whereas the Omani population appears much more admixed (Figure 2). Other populations exhibited different genetic make‐up, as indicated by their distinct genetic backgrounds. The heatmap of pairwise *F*_ST_ analyses (Table 2) illustrates that the lowest population differentiation is observed between the Ethiopian populations Alfa Midir and Negasi Amba (*F*_ST_ = 0.009), Gafera and Gesses (*F*_ST_ = 0.022), Gesses and Kido (*F*_ST_ = 0.022), and Gafera and Kido (*F*_ST_ = 0.024), as well as between the Chinese populations Dulong and Tibet (*F*_ST_ = 0.035). Conversely, the highest population differentiation was found between Chantecler and Fayoumi (*F*_ST_ = 0.349). Generally, there is moderate genetic differentiation between populations except for Fayoumi and Chantecler, which exhibit the highest *F*_ST_ values with the other populations.

**TABLE 2:** Pairwise population fixation (*F*_ST_) among chicken populations.

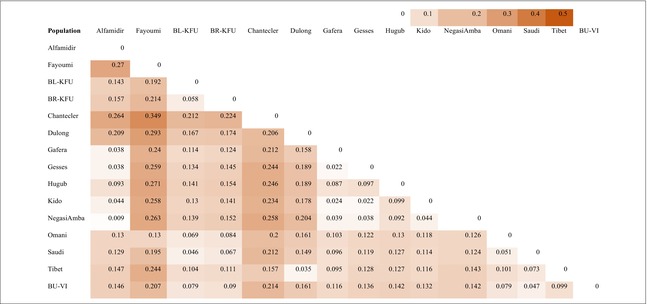

### Detecting signals of selective sweeps across the genome

Four different approaches were employed in selection tests to identify thermotolerance adaptations (heat and cold stress). We first aimed to identify candidate genomic regions potentially under positive selection for thermotolerance within each population separately. Population‐specific genomic regions were compared across groups to evaluate overlaps between hot‐climate populations (AP and Fayoumi) and cold‐climate populations (Chantecler, Dulong, and Tibet). The objective was to identify candidate regions showing significant differentiation in allele frequency (using the *ZF*_ST_ method) or in linkage disequilibrium patterns (using the *XP‐EHH* method). Candidate windows were identified through selection signature analysis with extreme 0.001 percentile, wherein the thresholds were set at standardized *F*_ST_ (*ZF*_ST_) >3.24–6.24, and absolute standardized *XP‐EHH* (|*XP‐EHH*_std|) >3.24–4.11.

### Candidate regions and genes associated with adaptation to extreme conditions within populations

**FIGURE 3:**
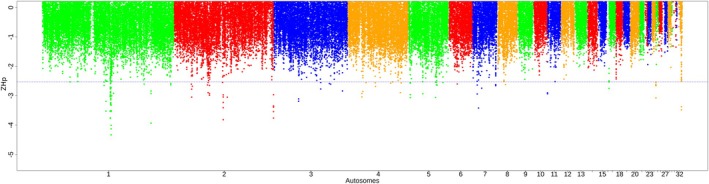
Example of Manhattan plot of the distribution of *ZHp* values (BL_KFU population). Additional figures are available in Figure S1a–l.

**FIGURE 4:**
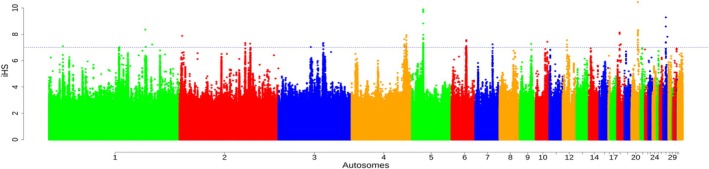
Example of Manhattan plot of the distribution of *iHS* values (BL‐KFU). Additional figures are available in Figure S2a–l.

We analyzed 81 007–95 110 and 95 101–95 122 windows across populations in *ZHp* and *iHS* analyses, respectively (Table S5). Significant *ZHp* sweeps were detected across the autosomes of the chicken genome, except on chromosomes 16, 19, 20, 21, 22, 25, 28, and 31 (Figure 3 and Table S6). *iHS* sweeps were observed across all autosomes except for chromosomes 25, 27, and 31 (Figure 4, Table S7). Across all AP populations under heat stress (Oman, Saudi Arabia, and Fayoumi), these windows collectively encompassed 169 genes (*ZHp*) and 172 genes (*iHS*) Table 3. Detailed results are provided in Tables S8a–n for *ZHp* and S9a–n for *iHS*, as well as in Figures S1a–l and S2a–n. The candidate genes exhibit different biological functions, particularly related to thermotolerance, stress response, immunity, and nervous system (Table S10). The analysis of GO terms and Kyoto Encyclopedia of Genes and Genomes (KEGG) pathways related to these genes provides additional insight into their potential importance for different biological pathways, including thermotolerance, adrenergic signaling in cardiomyocytes, response to bacterium, and protein phosphorylation (Tables S11 and S12). For instance, *RYR2* is implicated in stress‐induced polymorphic ventricular tachycardia, and interestingly, it was detected in Chinese populations under cold conditions (Wang et al., 2015). *LDB2* contributes to blood vessel formation (Javerzat et al., 2009). Additionally, *APP*, *NTN3*, and *PUF60* are associated with apoptotic responses to stress (Gu et al., 2020), while *CNTNAP2* plays roles in nervous system processes (von Holdt et al., 2023) were detected in the BL‐KFU population. In the AP populations, *ALX4* and *PLA2G15*, which play roles in fat deposition and fatty acid metabolic processes (McManus et al., 2022; Zhang et al., 2016), were detected along with *COL6A2*, a key gene in collagen synthesis (Fleming et al., 2017; Wang et al., 2016). Interestingly, the *COL6A2* gene was also detected in a genomic region displaying a substantial negative *ZHp* signal (−4.67) in Gesses and other Ethiopian populations, which reside in tropical warm/semiarid locations. Furthermore, *ORAOV1* is linked to resistance against oxidative stress, *FGF19* aids in metabolic adjustments during fasting, and *PLCE1* is potentially influencing higher intramuscular fat content.

**TABLE 3:** Common genes across most of the populations within genome regions showing extreme sweep signals (*iHS* ≥6.5 and Z*Hp* ≤−3.5).

CHR	Start (bp)	End (bp)	Method	Highest score	Genes and functions
5	17 070 001	17 140 000	*iHS*	8.24	*ORAOV1* is involved in the resistance to oxidative stress (Togashi et al., 2014)
					*FGF19* facilitates metabolic adjustments during fasting (Degirolamo et al., 2016)
6	20 920 001	20 940 000	*iHS*	8.42	*PLCE1* is involved in metabolism (Li et al., 2022)
1	102 900 001	102 920 001	*ZHp*	−4.28	*APP* regulates cell apoptosis (Gu et al., 2020)
2	149 040 001	149 060 001	*ZHp*	−3.90	*PUF60* is associated with apoptosis (Dong et al., 2019)
5	21 340 001	21 390 000	*ZHp*	−3.98	*ALX4* is associated with fat deposition (McManus et al., 2022)
11	50 001	70 000	*ZHp*	−3.76	*PLA2G15* is associated with fatty acid metabolic process (Zhang et al., 2016)
3	37 070 001	37 090 001	*ZHp*	−3.94	*RYR2* has a role in stress‐induced polymorphic ventricular tachycardia (Wang et al., 2015)
2	53 320 001	53 340 000	*ZHp*	−3.96	*CNTNAP2* roles is in the nervous system processes and may play a crucial role in how individuals adjust to novel environments (von Holdt et al., 2023)

Thirteen out of 133 genes detected in both *ZHp* and *iHS* may be related to adaptation to cold climate. A neurobehavioral‐relevant gene, *CNTNAP2*, was identified in the Chantecler population with the highest signal (*ZHp* = −3.76) within a region of 340 kb. *SRBD1* (S1 RNA binding protein 1) was identified across all populations residing in cold environments. It has been strongly associated with the induction of cell apoptosis (Ung et al., 2017). In Chinese populations, the lowest *ZHp* score was observed on Chromosome 24, which contains 10 different genes (*BCO2*, *TEX12*, *IL18*, *SDHD*, *PIH1D2*, *DLAT*, *C11ORF52*, *CRYAB*, *HSPB2*, and *DIXDC1*). The *STK38L* gene was identified in Chinese populations (*iHS* analysis) and is involved in neuronal cell division and morphology (Goldstein et al., 2010).

### Potential candidates and genes for heat‐stress adaptation comparing populations from hot‐arid and cold conditions

**FIGURE 5:**
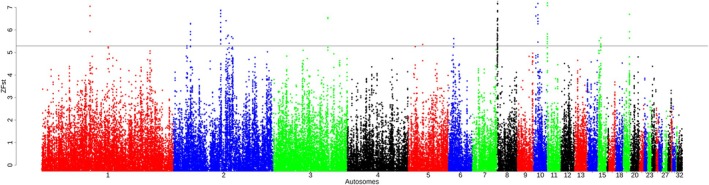
Example of Manhattan plot of the distribution of *ZF*_ST_ values in BL‐KFU vs. Chinese populations (Dulong and Tibet). Additional figures are available in Figures S3a–c and S4a–d.

**FIGURE 6:**
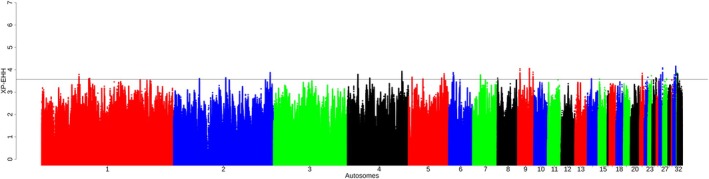
Example of Manhattan plot of the distribution of *XP‐EHH* values in BL‐KFU_versus_Chinese populations. Additional figures are available in Figures S6a–c and S7a–d.

**FIGURE 7:**
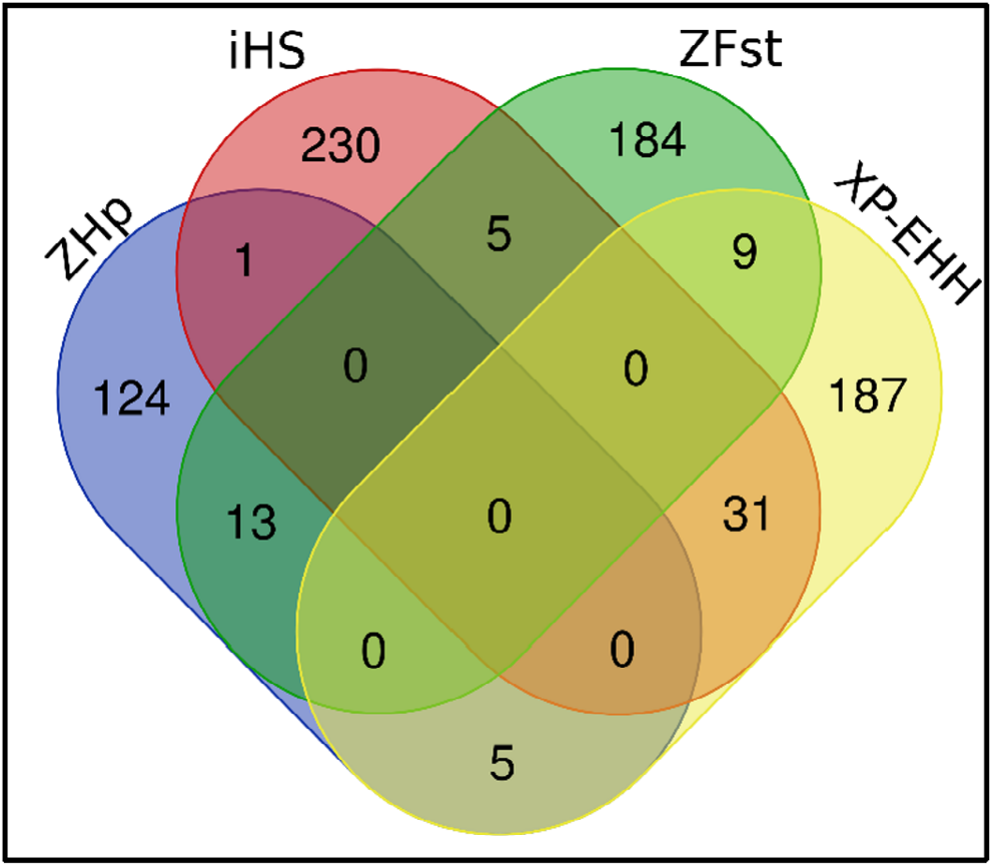
Venn diagram representing distribution of candidate genes based on *ZHp*, *iHS*, *ZF*_ST_ and *XP‐EHH* approaches across populations.

We analyzed 94 610 to 95 035 and 94 025 to 95 185 windows across populations by *ZF*_ST_ and *XP‐EHH* analysis, respectively (Table S13). To streamline the analysis, we only considered the overlapping candidate windows on the extreme 0.001 percentile of the empirical distribution. These include 392 and 336 sweep regions for *ZF*_ST_ and *XP‐EHH*, respectively, across all comparisons. Notably, significant *ZF*_ST_ sweeps were detected across the autosomes, except on chromosomes 16, 17, 18, 22, 23, 24, 25, 26, 27, 28, 31, 32, and 33 (Figure 5). *XP‐EHH* sweeps were observed across all autosomes except for chromosome 3 (Figure 6). These windows collectively encompass 226 genes (*ZF*_ST_ analysis) and 275 genes (*XP‐EHH* analysis) for the nine population pairwise comparisons from the hot‐arid condition (Oman, Saudi Arabia, and Fayoumi) compared to the cold environment (Chantecler, Dulong and Tibet) Table 4. Detailed results are provided in Table S14a–j and (Figures S3a–d and S4a–f) for *ZF*_ST_ and Table S15a–j and Figures S5a–c and S6a–d for *XP‐EHH*. The genes, identified using a combination of at least two methods (Table 5 and Figure 7), were found to be related to different environmental adaptation traits, including apoptosis, regulation of blood pressure, regulation of skin pigmentation, hypoxia, regulation of the equilibrium between energy storage and energy expenditure, metabolism, and immune responses. Five KEGG pathway terms related to different biological pathways: progesterone‐mediated oocyte maturation, adrenergic signaling in cardiomyocytes, GnRH signaling pathway, vascular smooth muscle contraction, and oocyte meiosis, were defined for these genes (Table S16).

**TABLE 4:** Genes within genome regions showing extreme sweep signals (*ZF*_ST_ >4 and *XP‐EHH* >3.5).

CHR	Start (bp)	End (bp)	Method	Highest score	Gene and functions	Comparisons
1	65 790 001	65 810 000	*ZF*_ST_	4.5	*SOX5*	BU‐VI vs. Chantecler
1	65 900 001	65 920 000	*ZF*_ST_	5.44	*SOX5*	Bl‐KFU vs. Chantecler
2	72 660 001	72 680 000	*ZF*_ST_	7.73	*CTNND2*	Omani vs. Chinese populations
3	50 260 001	50 280 000	*ZF*_ST_	5.28	*TFB1M*	Omani vs. Chantecler
5	45 420 001	45 440 000	*ZF*_ST_	4.11	*CLMN*	Fayoumi vs. Chantecler
5	4 120 001	4 140 000	*ZF*_ST_	6.39	*METTL15P1*	Fayoumi vs. Chinese populations
7	9 190 001	9 210 000	*ZF*_ST_	7.35	*SLC39A10*	Br‐KFU vs. Chinese populations
8	1 010 001	1 030 000	*ZF*_ST_	7.98	*VAV3*	Bl‐KFU vs. Chinese populations (Dulong and Tibet)
20	6 070 001	6 090 000	*ZF*_ST_	5.35	*EYA2*	Br‐KFU vs. Chantecler
18	11 300 001	11 330 000	*XP‐EHH*	5.34	*SUMO2 and NUP85*	BU‐VI vs. Chantecler
24	6 290 001	6 310 000	*XP‐EHH*	4.84	*C11ORF52*, *CRYAB*, *HSPB2*, *C11orf1*, *FDXACB1*, and *ALG9*	Bl‐KFU vs. Chantecler
16	620 001	650 000	*XP‐EHH*	4.58	*5_8S_rRNA*	Omani vs. Chinese populations (Dulong and Tibet)
24	6 290 001	6 320 000	*XP‐EHH*	4.55	*C11ORF52*, *CRYAB*, *HSPB2*, *C11orf1*, *FDXACB1*, and *ALG9*	Omani vs. Chantecler
14	250 001	280 000	*XP‐EHH*	3.90	*CPPED1*	Fayoumi vs. Chantecler
12	14 590 001	14 620 000	*XP‐EHH*	3.98	*SLC25A26 and LRIG1*	Br‐KFU vs. Chinese populations (Dulong and Tibet)
9	18 070 001	18 090 000	*XP‐EHH*	4.10	*NAALADL2*	Bl‐KFU vs. Chinese populations (Dulong and Tibet)
28	5 050 001	5 100 000	*XP‐EHH*	5.14	*PTPRS*	Br‐KFU vs. Chantecler

**TABLE 5:** Candidate regions and genes linked to environmental adaptation identified within and between chicken populations analyses.

Chr	Chromosomal region	Analysis		Gene	Functions
4	75 820 001–75 850 000	BL‐KFU vs. Chantecler (*ZF*_ST_), Br‐KFU vs. Chantecler (*ZF*_ST_), Omani vs. Chantecler (*ZF*_ST_), Omani vs. Chinese populations (*ZF*_ST_)	Chinese populations (*ZHp*), Br‐KFU (*ZHp*), BU‐VI (*ZHp*), and Fayoumi (*ZHp*)	*LDB2*	Involved in brain development, blood vessel formation (Javerzat et al., 2009)
5	3 490 001–3 510 000	Fayoumi vs. Chantecler (*ZF*_ST_) and Fayoumi vs. Chinese populations (*ZF*_ST_)	BU‐VI (*iHS*)	*ANO3*	Associated with metabolism (Jean et al., 2015)
6	17 400 001–17 430 000	Br‐KFU_vs. Chinese populations (*ZF*_ST_), Br‐KFU. vs. Chinese populations (*XP‐EHH*), and Br‐KFU vs. Chantecler (*ZF*_ST_)		*ITGA6*	Linked directly regulated by hypoxia‐inducible factors (Brooks et al., 2016)
7	6 670 001–6 710 000	Br‐KFU vs. Chinese populations (*ZF*_ST_), Br‐KFU vs. Chantecler (*ZF*_ST_)	BL‐KFU (*ZHp*) and Fayoumi (*ZHp*)	*COL6A2*	Involved in collagen biosynthesis and enzyme modification (Fleming et al., 2017; Wang et al., 2016)
14	280 001–310 000	Br‐KFU vs. Chantecler (*XP‐EHH*), Fayoumi vs. Chantecler (*XP‐EHH*), and Omani vs. Chantecler (*XP‐EHH*)	BU‐VI (*iHS*), and Fayoumi (*iHS*)	*CPPED1*	Associated with immune system processes (Bentz et al., 2019; Chen et al., 2022)
15	3 390 001–3 420 000	BL‐KFU vs. Chantecler (*ZF*_ST_)	Chinese populations (*ZHp*)	*FZD10*	Involved in regulating skin pigmentation, a critical aspect of protection against solar radiation. (Nie et al., 2022)
18	3 830 001–3 870 000	BL‐KFU vs. Chinese populations (*XP‐EHH*), and BL‐KFU vs. Chantecler (*ZF*_ST_)		*CASZ1*	Played a crucial role in regulating blood pressure (Simino et al., 2014)
19	9 350 001–9 410 000	BL‐KFU vs. Chantecler (*ZF*_ST_), and BU‐VI vs. Chantecler (*XP‐EHH*)		*MSI2*	Involved in the apoptosis process (Bennett et al., 2016)
23	5 950 001–5 980 000	Omani vs. Chantecler (*XP‐EHH*)	BR‐KFU (*iHS*).	*PNRC2*	Involved in regulating the equilibrium between energy storage and energy expenditure (Sorbolini et al., 2017)
				*SRSF10*	May be involved in promoting cell survival during stressful conditions (Zhou et al., 2014)
24	6 290 001–6 320 000	BU_VI vs. Chantecler (*XP‐EHH*), Bl‐KFU vs. Chantecler (*XP‐EHH*), Br‐KFU vs. Chantecler (*XP‐EHH*), and Omani vs. Chantecler (*XP‐EHH*)	Chantecler (*iHS*)	*ALG9*	Associated with lipid metabolism (Iqbal et al., 2023)

The most significant peaks are on chromosomes 2, 4, 7, 8, 11, and 19 in the *ZF*_ST_ analyses (all nine comparisons). These candidate regions include *VAV3* linked to hypoxia, previously reported in Ethiopian chickens (Scheinfeldt et al., 2012). Other interesting genes are: *NTNG1*, involved in secretion, is significantly up‐regulated by heat stress in the male White Leghorn chicken hepatocellular cell line (Sun et al., 2015); *VSTM2A* related to behavior (Wang et al., 2023); *SMPD3* and *EYA2* linked to growth performance (Bounas et al., 2023; Feng et al., 2023); *DYNLL2* to the immune responses (Ogada et al., 2023); *SLC39A10* to metabolism (Zhu et al., 2019); *COL18A1* to collagen biosynthesis and enzyme modification (Wang et al., 2016); and *TG* thyroglobulin known to affect the ability to accumulate intramuscular fat (Kostusiak et al., 2023). One gene related to blood circulation is *SOX5*, which is crucial in redirecting blood flow to the skin and facilitating heat exchange during high temperatures (Hester et al., 2015).

As for the *XP‐EHH* results, we found within candidate genome regions *SLC25A26*, *LRIG1*, and *JADE3*. Also present are heat shock protein family B (small) member 2 (*HSPB2*) and crystallin α B (*CRYAB*) involved in stress tolerance, muscle cell integrity, and cell survival against heat stress (Du et al., 2022; Nakagawa et al., 2001; Wang et al., 2016), *ALG9* (lipid metabolism) (Iqbal et al., 2023), *PTPRS* (insulin signaling and inflammatory pathways) (Samblas et al., 2018), and *NUP85* (stress responses) (Zhu et al., 2017).

## DISCUSSION

### Diversity and population structure

This study is the first large‐scale whole genome sequence analysis of indigenous chickens from the AP, covering eastern Saudi Arabia and Oman. The main aim was to assess genetic diversity and the adaptive landscape of the genome of indigenous chickens from the AP, in response to extreme thermal challenges (hot and arid climates), contrasting these populations with other chicken populations known for their adaptation to harsh cold environmental conditions.

We used the chicken genome reference GRCg6a to call genome‐wide SNPs for 156 chickens, identifying 24.9 million SNPs, of which 38.24% were novel. Previous studies by Lawal et al. (2018) and Gheyas et al. (2022) reported 10 million (16% novel) and 19.5 million (29% novel) SNPs in domestic chickens of Ethiopia, respectively. A possible explanation for these differences includes a different version of the chicken reference genome, the number of samples, and the difference in filter metrics and genome coverage. The average SNP density, with a value of 26.49 SNPs/kb, surpasses the previously reported density of 15 SNPs/kb in experimental and commercial chicken populations based on the galGal5 reference genome (Gheyas et al., 2015). The SNP density in Ethiopian indigenous chicken populations, using galGal6 as a reference genome, was approximately 19 SNPs/kb in Gheyas et al. (2022) study. Disparity suggests that improvements in genome assembly quality may have contributed to the observed increase in SNP density in our study.

PC and admixture analyses showed genetic differentiations between the populations and separated most of them based on their geographic areas of origin. A relatively proximity was observed between AP and Fayoumi chicken supporting a shared genetic heritage between these two lines, which are highly adapted to heat stress, unlike other populations. Interestingly, Chantecler chicken are found relatively close to the local Chinese populations (Dulong and Tibet) despite their very distinct geographic origins, which were separated into different clusters based on the admixture analysis. These results suggest that most of our studied populations may not have been traded or historically intermixed with others for a long time. The exception might be the Omani population, which shows a high level of admixture from different genetic backgrounds, probably a legacy of multiple introductions along the eastern coast of the AP.

### Genes under positive selection associated with heat tolerance

The harsh desert climate of the AP, characterized by scorching temperatures and arid conditions due to intense solar radiation, presents a significant challenge for living organisms, including chickens. In response to these extreme conditions, chickens have developed a range of strategies to maintain their body temperature. These strategies involve using enhanced radiation, convective heat dissipation, and evaporative cooling through their diastolic blood pressure. Notably, chickens are referred to as “true panting animals”, indicating that they can release internal heat by evaporating moisture through their lungs and air sacs. This panting mechanism serves as a crucial tool for regulating body temperature in the face of relentless heat (Collier & Gebremedhin, 2015; Lara & Rostagno, 2013; Mutaf et al., 2009). Therefore, by scrutinizing the results of four different methods for detecting candidate positive signatures of selection and investigating the roles of genes within these regions, we may be able to link those to specific thermotolerance strategies.

In this study, we identified several genes associated with the cardiovascular system development and blood vessel formation (*RYR2*, *LDB2*, *SOX5*, and *FHOD3*) that may have been under positive selection in the chicken populations from the AP. Notable examples include *RYR2*, known for its association with stress‐induced polymorphic ventricular tachycardia and which may be associated with adaptation to high altitude in the Tibetan chickens (Wang et al., 2015). Other genes with important functions are *LDB2*, which plays a pivotal role in blood vessel formation (Javerzat et al., 2009), and *SOX5*, known in Leghorn chicken to reroute blood flow to the skin, facilitating heat exchange (Hester et al., 2015). Also worth mentioning is *FHOD3*, belonging to the formin family of proteins, which has a pivotal role in cardiac development (Rosado et al., 2014). *FHOD3* is prominently expressed in the heart and plays a vital role in myofibrillogenesis, particularly in myofibril maturation (Kan‐o et al., 2012). All these genes may be closely associated with the regulation of blood flow and evaporative cooling mechanisms.

We have also identified significant enrichment of functional pathways following KEGG analysis (*p* < 0.05). It includes the adrenergic signaling pathway in cardiomyocytes. This finding is of particular importance because adrenergic signaling is a central regulator of cardiac function, allowing these birds to dynamically adjust their heart rate and cardiovascular responses in response to the changing environmental conditions they encounter. Four genes (*PRKCD*, *BRAF*, *ADCY7*, and *ADCY6*), part of the vascular smooth muscle contraction pathway, were also identified. This pathway is involved in the control of the systolic and diastolic functions responding to varying physiological demands (Schaub et al., 2006). Interestingly, some of these results align with previous research conducted in Saudi Arabian local chickens by Tian et al. (2020), which also emphasized the role of the adrenergic and muscarinic systems in controlling heart rate and cardiac function in response to diverse physiological challenges.

The presence of genes associated with apoptosis is a significant finding in this study. Heat stress can lead to cellular injury in the liver, heart, kidneys, and central nervous system, emphasizing the importance of understanding the genetic basis of apoptosis regulation in response to environmental stressors. Apoptosis is a highly regulated and controlled cell death process crucial for maintaining internal stability in living organisms and facilitating adaptation to the surrounding environment (Elmore, 2007). In the context of the AP's challenging environmental conditions, this finding carries particular significance. One of the identified genes, *SRBD1*, is known to play a role in cell growth and the regulation of apoptosis (Mizuki, 2010). It was related to the adaptation to the driest and warmest environments of wild great tit bird populations (Stonehouse et al., 2024). Moreover, another study has emphasized the presence of genes involved in cell differentiation and apoptosis in neural cells, such as *APP* and *PUF60* in Chinese native chickens (Gu et al., 2020). The *SLC26A8* gene is also noted, potentially playing a role in cell regulation or apoptosis (Dirami et al., 2013). Another important finding is the reference to the death‐associated protein (*DAP*) gene, which is associated with the regulation of myogenesis, apoptosis, and skeletal development in chickens (Tesseraud et al., 2021). Interestingly, we also identified an enrichment pathway related to apoptosis in the Chantecler and Chinese populations that live in cold environments.

The presence and function of melanin pigment (skin, feathers) are crucial for shielding animals from the damaging effects of solar radiation. Wnt signaling is crucial in regulating epidermal stratification, essential for maintaining skin health and function. Within the context of this study, seven candidates positively selection genes were identified in the melanogenesis pathway (*POMC*, *MAP2K2*, *FZD5*, *CREB3L3*, *WNT5B*, *FZD10*, and *CALM1*). Among these genes, three, namely *WNT5B*, *FZD5*, and *FZD10*, are associated with Wnt signaling pathway, which includes also genes such as *PPP3CA*, *CCND2*, *CTNND2*, *LGR6*, *SIRT1*, *WISP1*, and *LGR4*. The Wnt signaling pathway, known to induce epidermal stratification and regeneration (Zhu et al., 2014), shows significant enrichment in AP camels, which may be related to their adaptation to desert environments (Al Abri et al., 2023; Bahbahani et al., 2024). Specifically, the *FZD10* gene appears to have a connection with melanin formation (Nie et al., 2022), suggesting its potential role in regulating pigmentation, a critical aspect of protection against solar radiation. Another gene of interest is *BCO2*, which has been linked to the process of pigmentation (Wu et al., 2021). Its presence in AP populations underscores its significance in skin and feathers adaptation to the intense solar radiation of the region. Our study also identified in AP population a candidate signature of positive selection in a region including *SIRT1*. This observation suggests that the genetic traits of these populations are responsive to solar radiation (Tian et al., 2020). *SIRT1* is recognized for its role in various cellular processes, including DNA repair and the regulation of oxidative stress responses, which are crucial for reducing the damage from solar radiation exposure. These findings support that these populations are adapted to minimize the negative impact of solar radiations.

## CONCLUSION

This study documents the genetic diversity, population structure and adaptations of AP indigenous chicken populations to harsh climatic environments in a national and global context. Our results show the clustering of all populations according to their geographic region of origin, with minimal genetic differentiation observed within the populations. To the best of our knowledge, this is the first large‐scale whole genome sequence analysis that targets the indigenous chickens from the AP, covering eastern Saudi Arabia and Oman.

Considering the well‐documented interaction between the evolution of a species and environmental factors, this study also explores the genetic signature of positive selection in populations adapted to different temperature ranges across various geographic locations. Our investigation also involved the identification of numerous genes associated with environmental adaptation within each population, as well as the differentiation of gene profiles between these populations. The study unveiled a range of genes related to diverse functions, including the regulation of metabolism and energy (*SUGCT*, *HECW1*, *MMADHC*), cardiovascular system development and angiogenesis (*RYR2*, *LDB2*, *SOX5*, *FHOD3*), apoptosis in cells (*APP*, *SRBD1*, *NTN1*, *PUF60*, *SLC26A8*, *DAP*, *SUGCT*), protection against solar radiation (*FZD10*, *BCO2*, *WNT5B*, *COL6A2*, *SIRT1*), and growth (*NELL1*). These genes may play a role in the adaptation to thermal stress, potentially with different alleles or haplotypes contributing to adaptation to either cold or heat stress. However, it is important to emphasize that further investigations are warranted to delve deeper into these findings. Still, our results provide valuable support for the presence of genetic adaptations among the studied populations adapted to different environmental conditions.

## AUTHOR CONTRIBUTIONS

**Abdulwahab Assiri:** Conceptualization, data analysis, writing – original draft, sample collection, DNA extraction. **Olivier Hanotte:** Project administration, supervision, writing – review & editing. **Hussain Bahbahani:** Data analysis, writing – review & editing. **Mohammed Al‐Abri:** Sample collection, DNA extraction. **Waleed Al Marzooqi:** Sample collection, DNA extraction. **Faisal Almathen:** Supervision, data analysis. **Adriana Vallejo‐Trujillo:** Data analysis, writing – review & editing. **Abulgasim Ahbara:** Data analysis, writing – review & editing. **Raman Lawal:** Data analysis. **Abdulfatai Tijjani:** Data analysis.

## CONFLICT OF INTEREST STATEMENT

The authors declare that they have no competing interests.

## Supporting information


Appendix S1.

Appendix S2.

## Data Availability

The paired‐read Illumina fastq files of the new genome samples are available in the European Nucleotide Archive under project PRJEB77573.
