# Supplementary material for: Comparative genomics reveals common diversity and adaptation to harsh environments in the Arabian Peninsula indigenous chickens
Source: Anim Genet. 2025 May 2;56(3):e70014. doi: 10.1111/age.70014 (PMC12046372; doi:10.1111/age.70014)
Supplement: Supplementary file 1 — Appendix S1. [file AGE-56-0-s001.pdf]

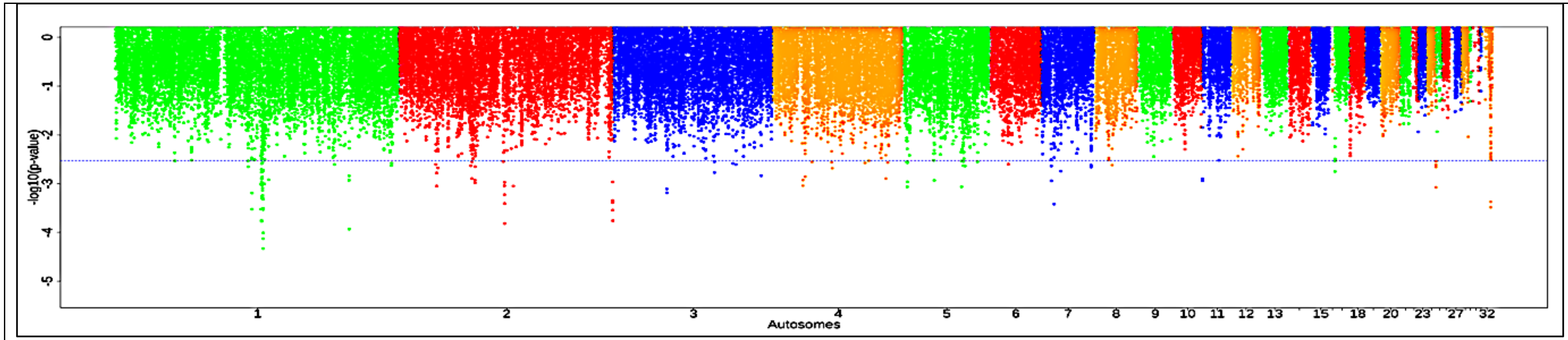

(a)

Manhattan plot of the distribution of *ZHp* values in **BL-KFU.** The horizontal dashed line indicates the threshold significant at *ZHp* =~ -2.60

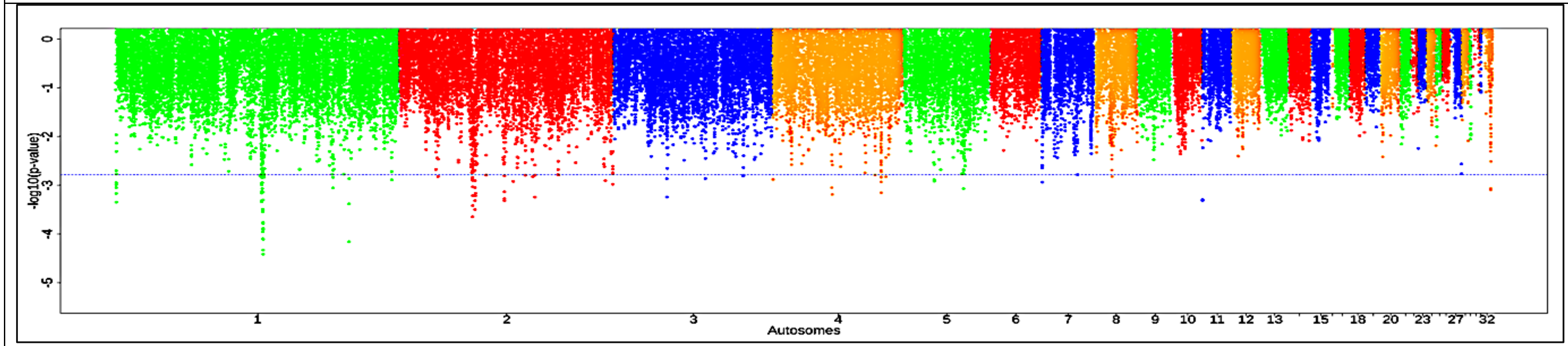

(b)

Manhattan plot of the distribution of *ZHp* values in **BR-KFU**. The horizontal dashed line indicates the threshold significant at *ZHp* =~ -2.78

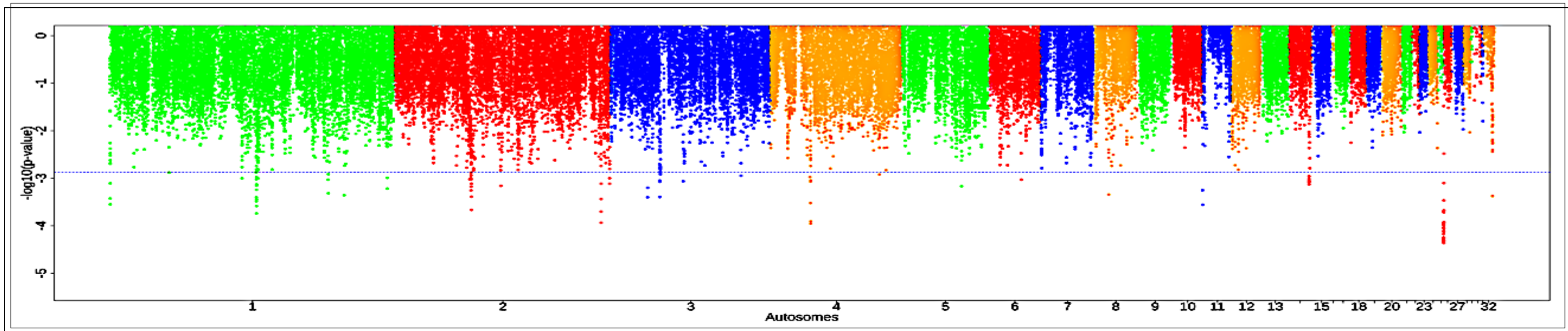

(c)

Manhattan plot of the distribution of *ZHp* values in **BU-VI**. The horizontal dashed line indicates the threshold significant at *ZHp* =~ -2.87

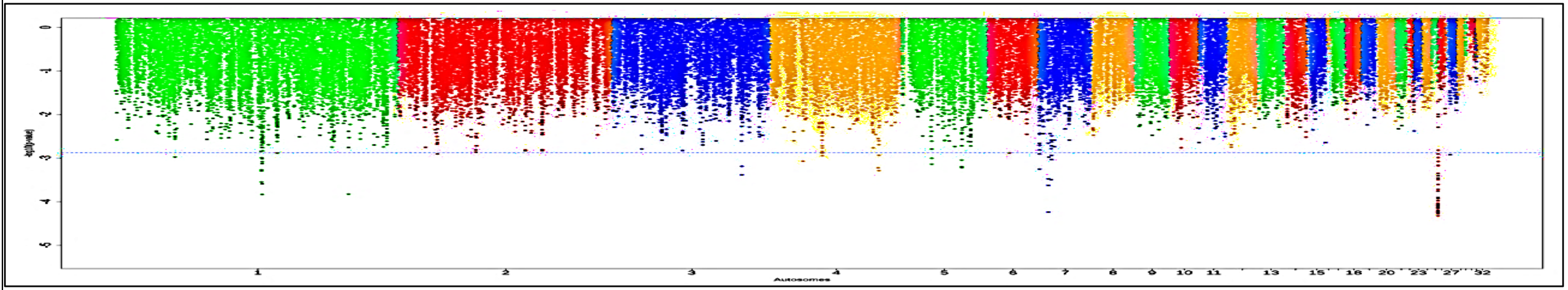

(d)

Manhattan plot of the distribution of *ZHp* values in **Fayoumi.** The horizontal dashed line indicates the threshold significant at *ZHp* =~ -2.74

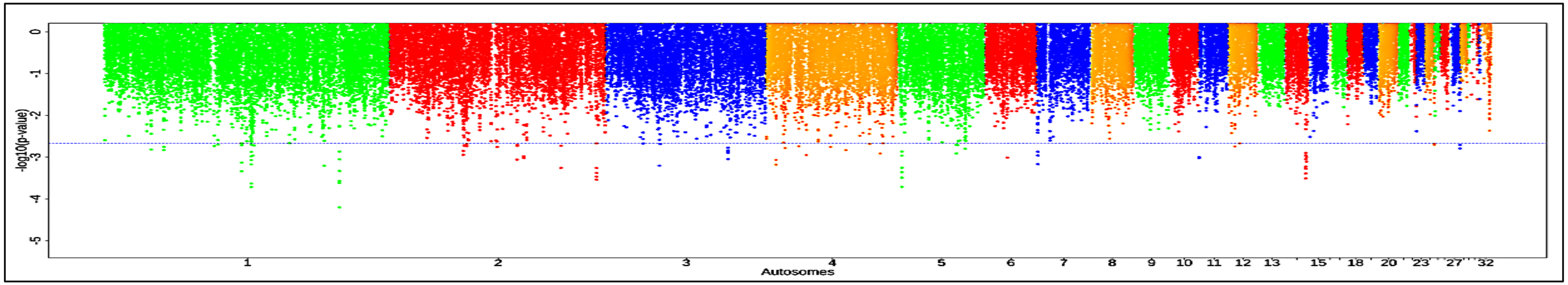

(e)

Manhattan plot of the distribution of *ZHp* values in **Oman.** The horizontal dashed line indicates the threshold significant at *ZHp* =~ -2.66

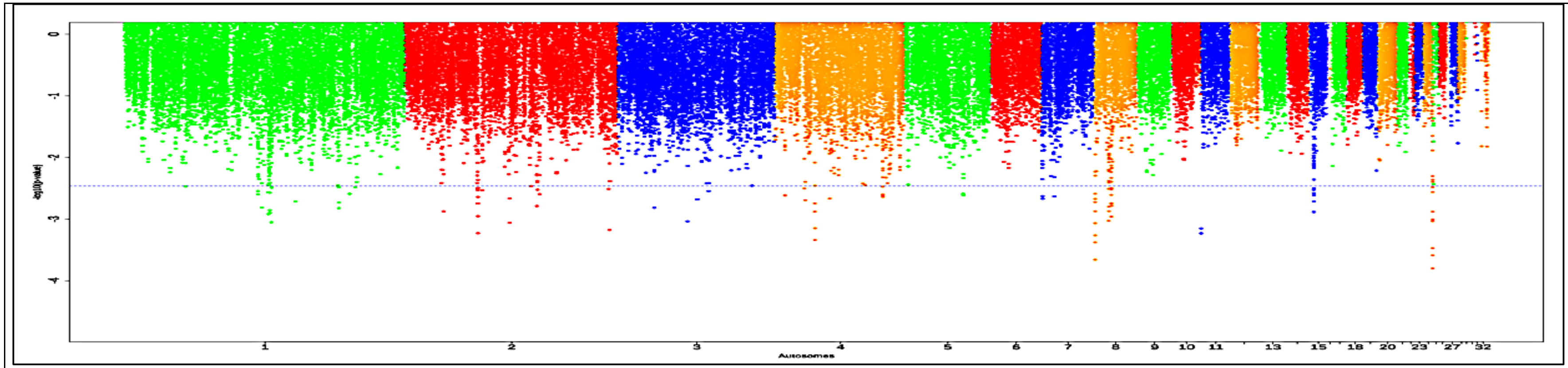

(f)

Manhattan plot of the distribution of *ZHp* values in DT-China (**Dulong and Tibet).** The horizontal dashed line indicates the threshold significant at *ZHp* =~ -2.49

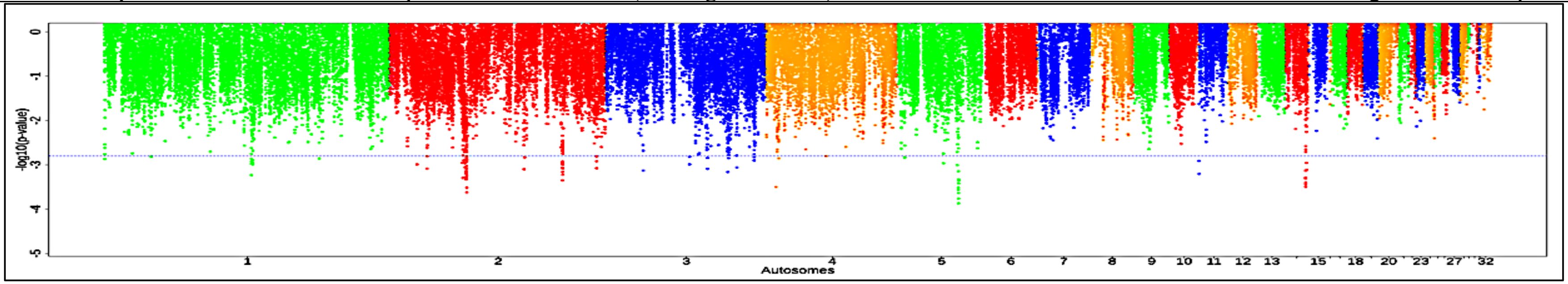

(g)

Manhattan plot of the distribution of *ZHp* values in **Chantecler.** The horizontal dashed line indicates the threshold significant at *ZHp* =~ -2.80

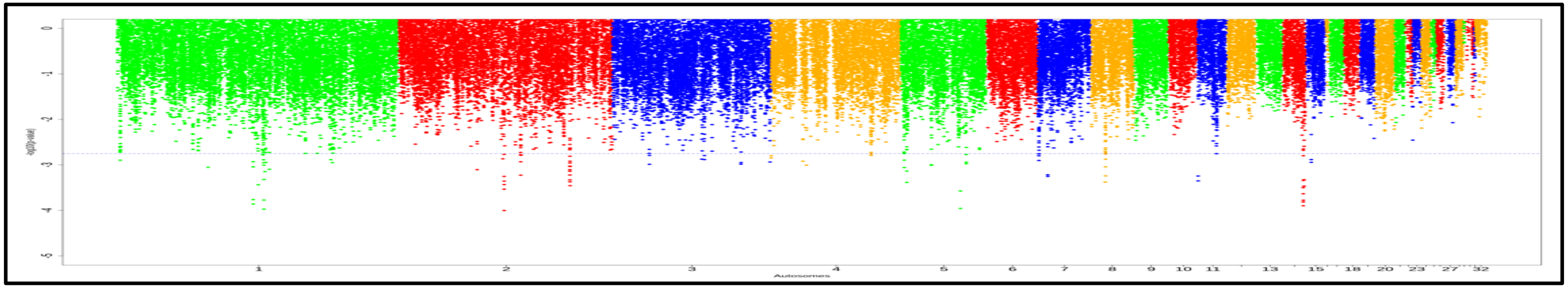

(h)

Manhattan plot of the distribution of *ZHp* values in **Alfa_midir.** The horizontal dashed line indicates the threshold significant at *ZHp* =~ -3.82

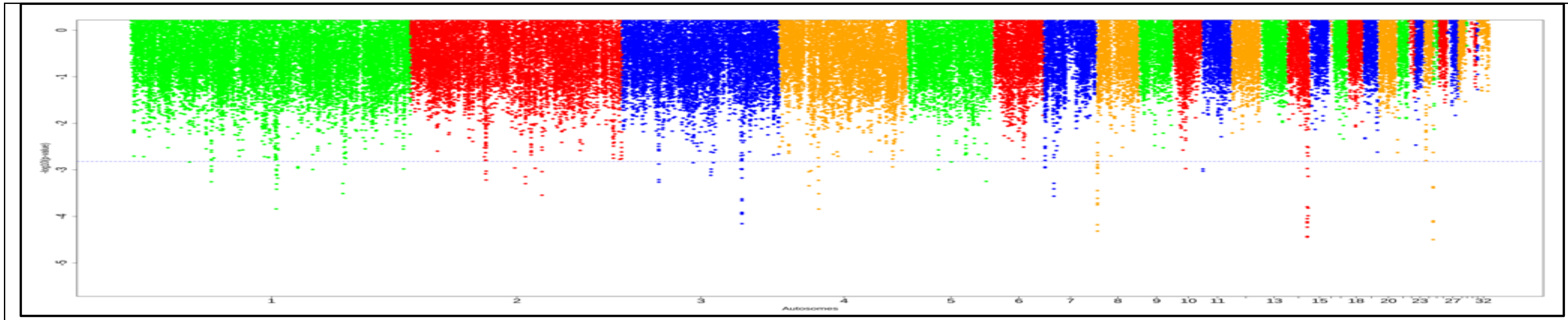

(i)

Manhattan plot of the distribution of *ZHp* values in **Gafera.** The horizontal dashed line indicates the threshold significant at *ZHp* =~ -2.86

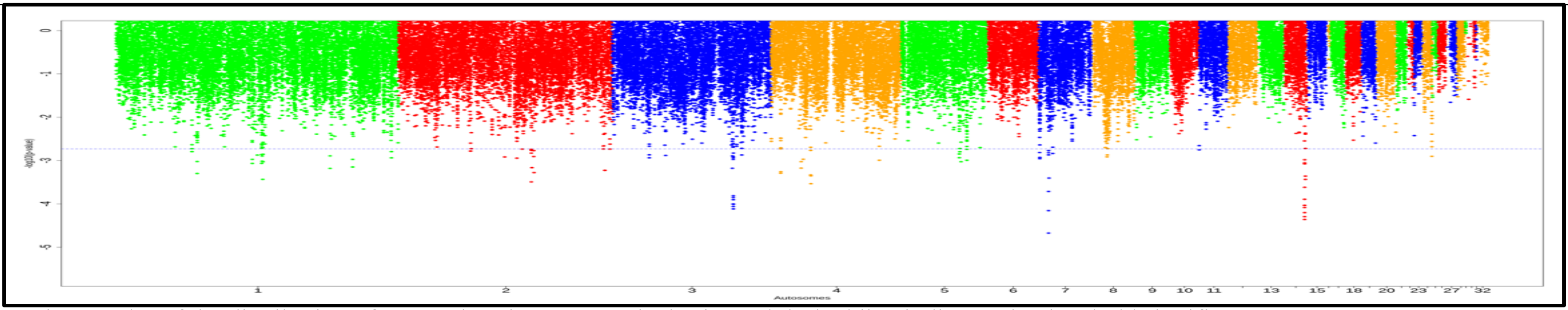

(j)

Manhattan plot of the distribution of *ZHp* values in **Gesses.** The horizontal dashed line indicates the threshold significant at *ZHp* =~ -2.90

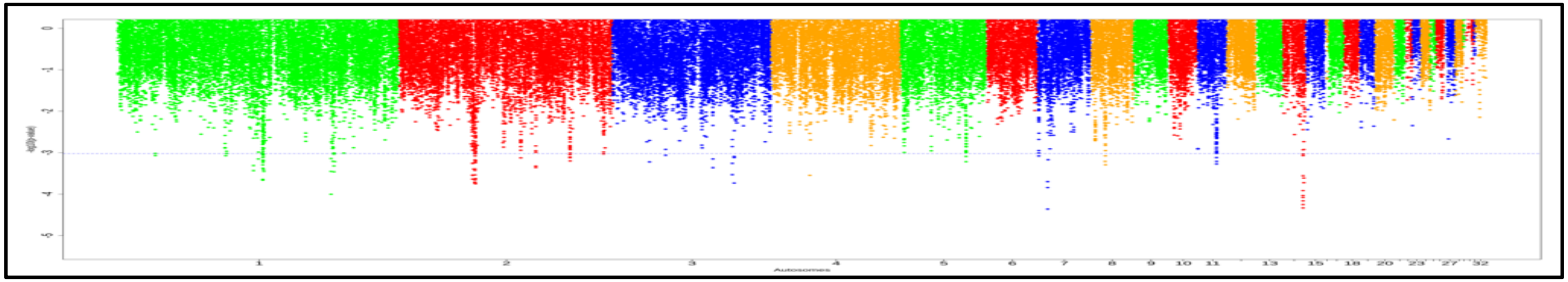

(k)

Manhattan plot of the distribution of *ZHp* values in **NegasiAmba.** The horizontal dashed line indicates the threshold significant at *ZHp* =~ -3.03

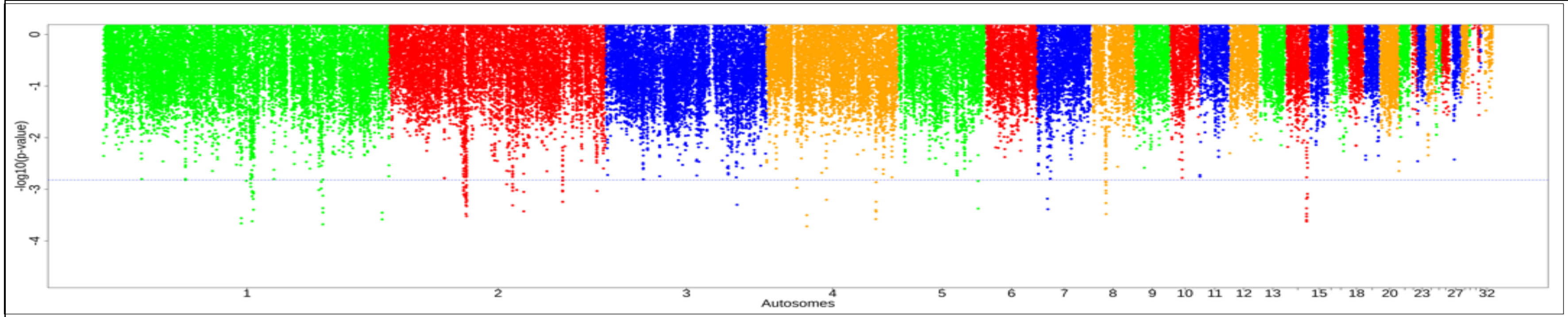

(l)

Manhattan plot of the distribution of *ZHp* values in **Kido.** The horizontal dashed line indicates the threshold significant at *ZHp* =~ -2.9

Fig S1. Manhattan plot of the distribution of *ZHp* values for all selected populations. The dashed horizontal lines represent the cut-off for extreme *ZHp* scores, corresponding to the upper 0.001 percentile of *ZHp* values.

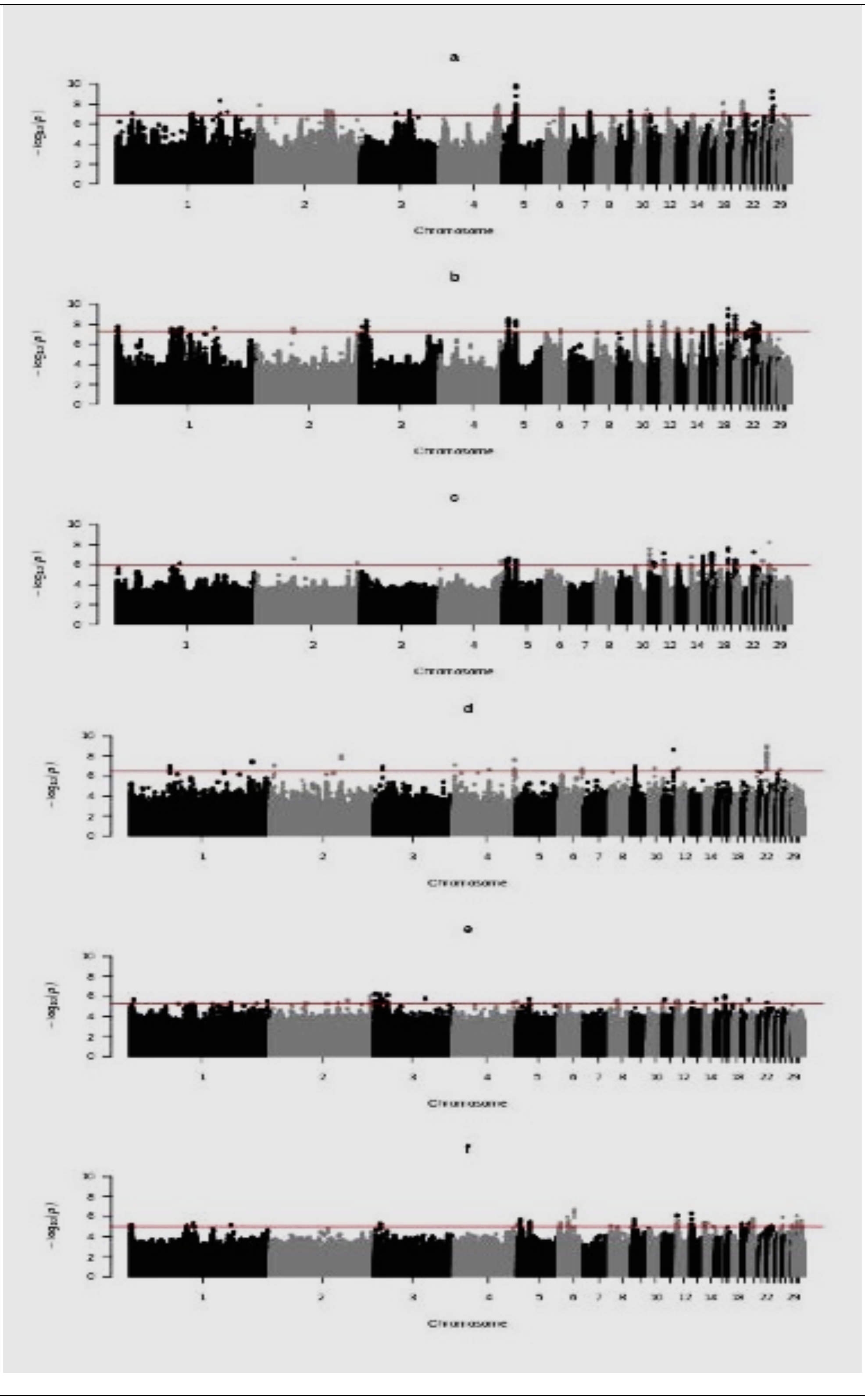
a
b
c
d
e
f
Chromosome

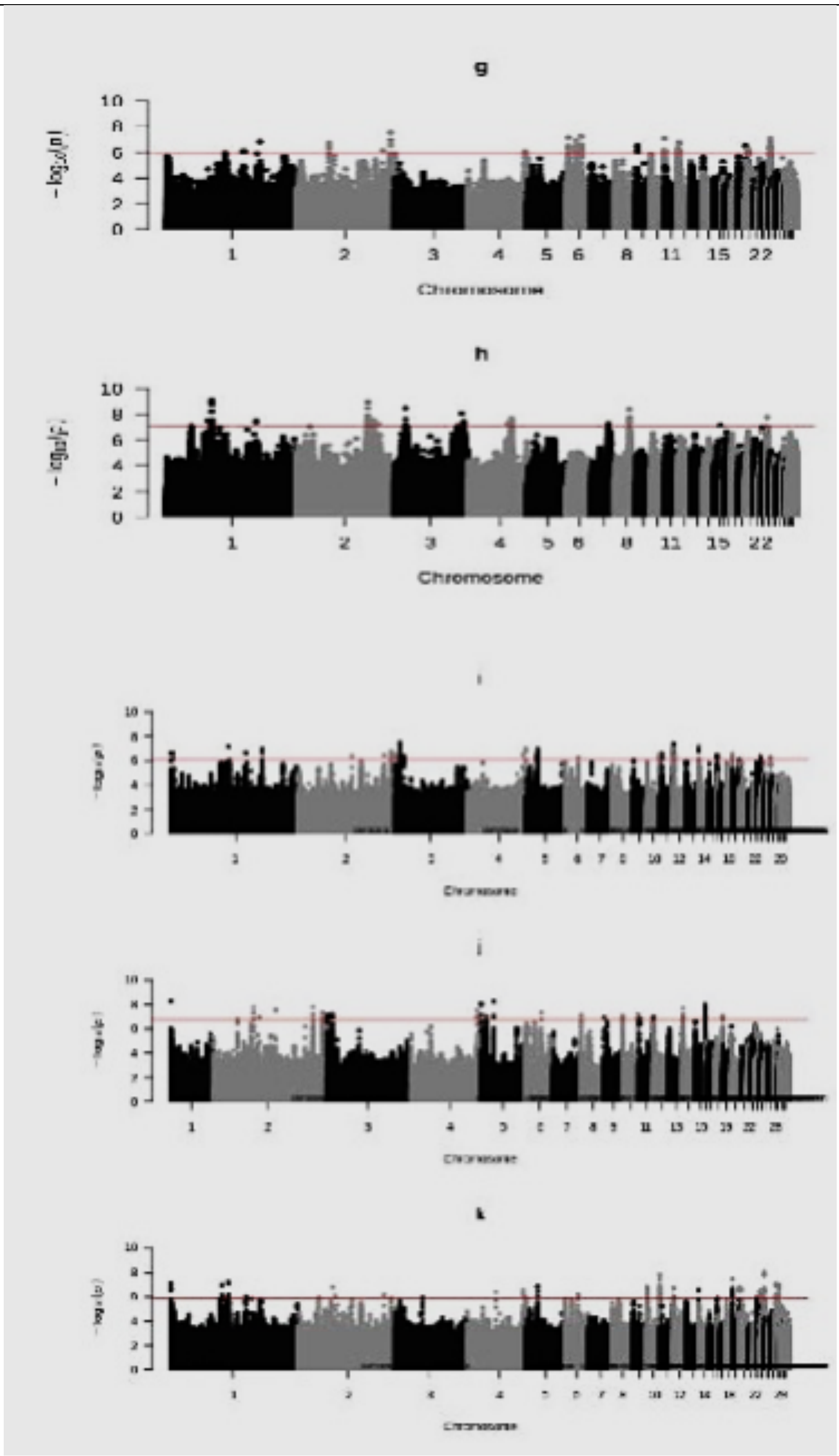
g
h
j
k
Chromosome

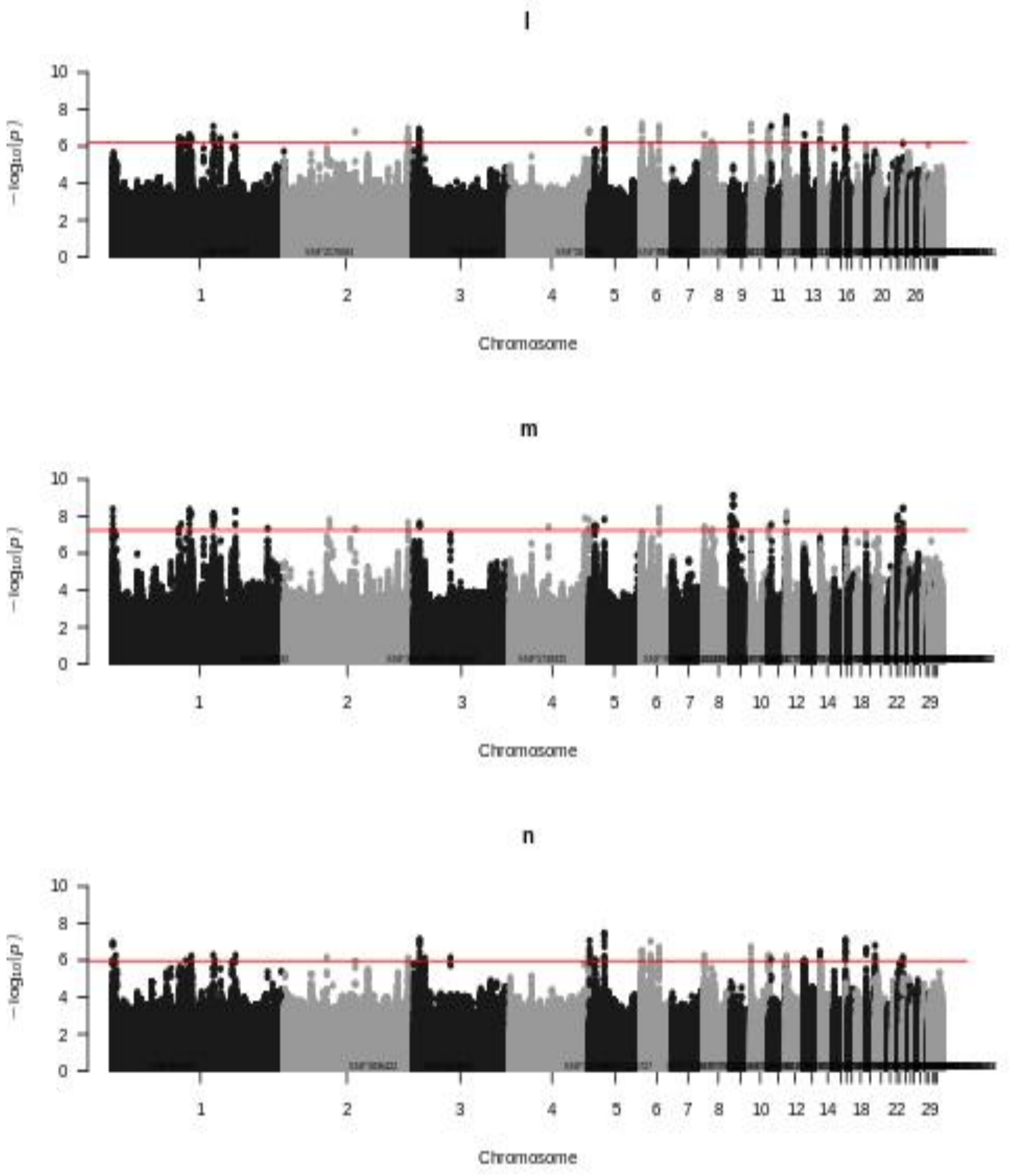

Fig S2 Integrated haplotype score (*iHS*) plots for selected populations. (a–n) Manhattan plots of standardized *iHS* statistics for selected populations in (a) BL-KFU, (b) BR-KFU, (c) BU-VI, (d) Omani, (e) Fayoumi, (f) Saudi, (g) Chantecler, (h) Chinese (Dulong and Tibet), (i) Gesses, (j) Hugub, (k) Kido, (l) NegasiAmba, (m) Gafera, and (n) Alfa Madir. The dashed horizontal lines represent the cut-off for extreme *iHS* scores, corresponding to the upper 0.001 percentile of *iHS* values.

(a)

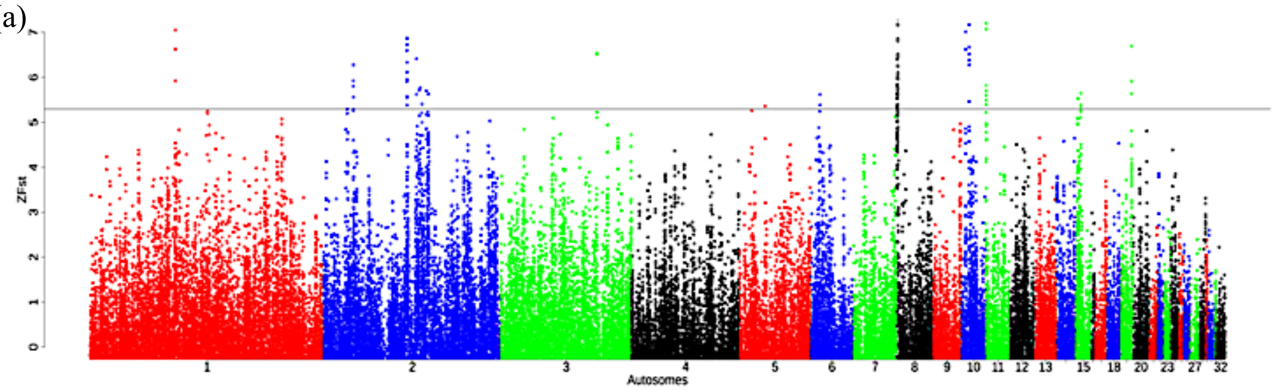

Manhattan plot of the distribution of ZF_{ST} values in **BL-KFU vs. DT-China.**

(b)

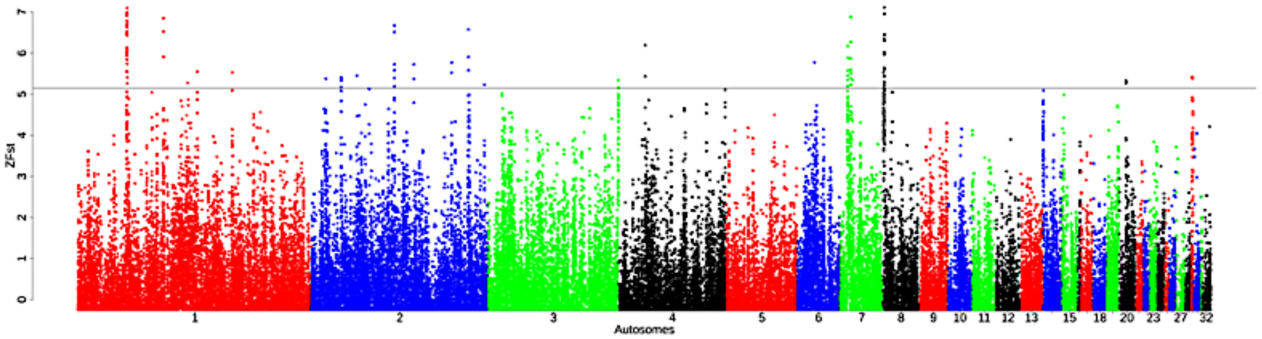

Manhattan plot of the distribution of ZF_{ST} values in **Br-KFU vs. DT-China.** The horizontal dashed line indicates the threshold significant at *ZFST* =~ 5.10

(c)

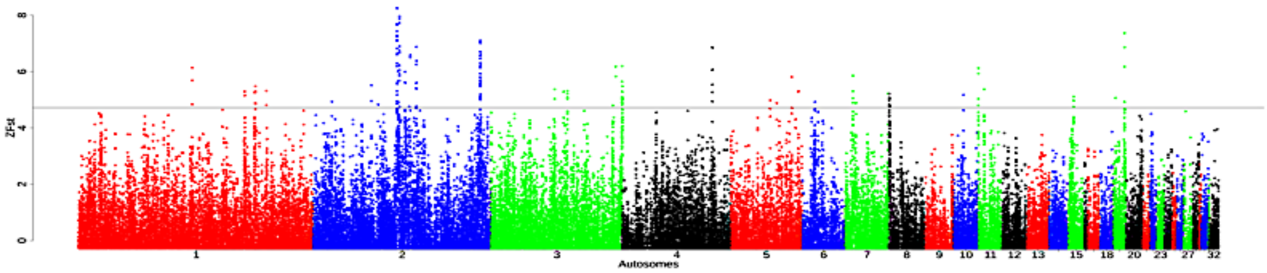

Manhattan plot of the distribution of ZF_{ST} values in **Omani vs. DT-China.**

(d)

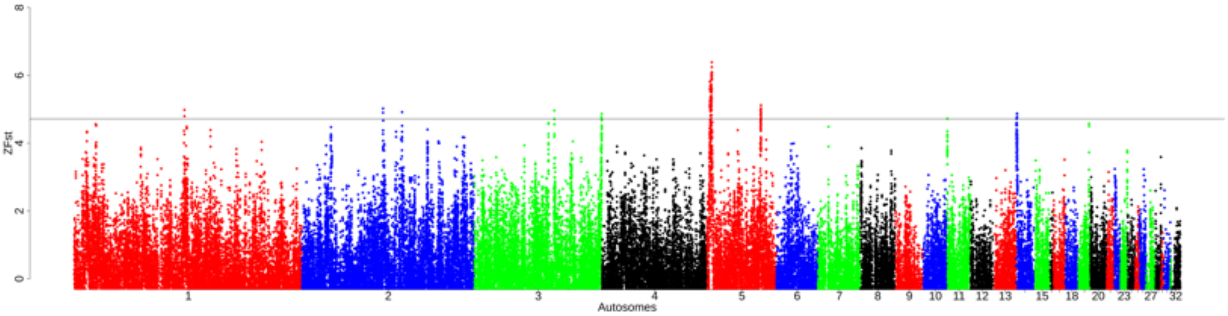

Manhattan plot of the distribution of ZF_{ST} values in **Fayoumi vs. DT-China.** The horizontal dashed line indicates the threshold significant at ZF_{ST} =~ 4.39

Fig S3. ZF_{ST} plots for comparisons. (a–d) Manhattan plots of standardized ZF_{ST} statistics for (**Arabian Peninsula populations vs. DT-China) and (Fayoumi vs. DT-China)** comparisons. The dashed horizontal lines represent the cut-off for extreme ZF_{ST} scores, corresponding to the upper 0.001 percentile of ZF_{ST} values.

(a)

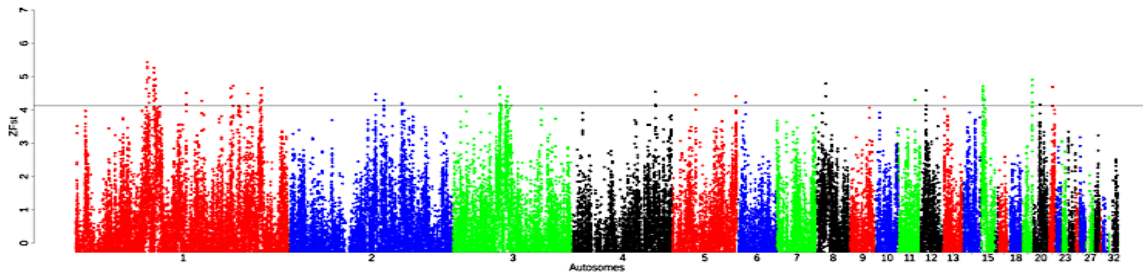

Manhattan plot of the distribution of *ZFST* values in **Bl-KFU vs. Chantecler.**

(b)

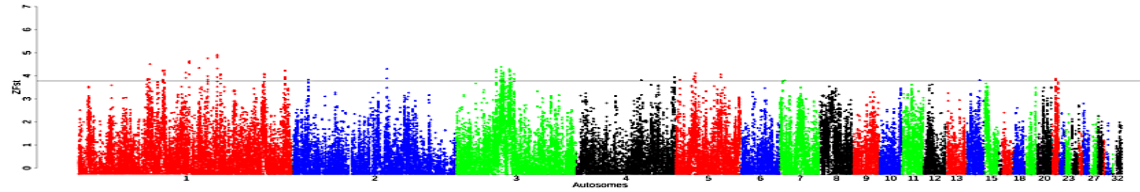

Manhattan plot of the distribution of *ZFST* values in **BU-VI vs. Chantecler.**

(c)

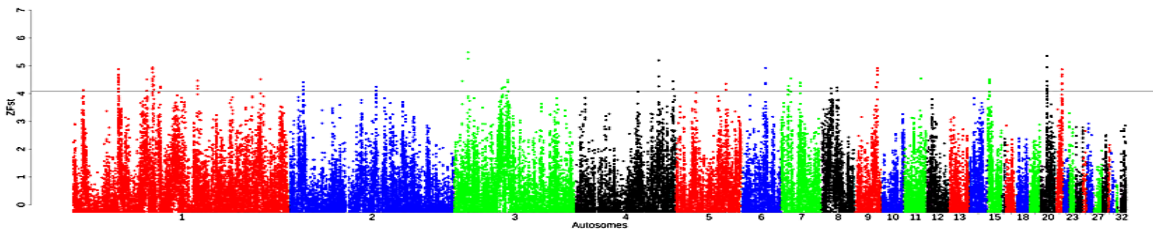

Manhattan plot of the distribution of *ZFST* values in **Br-KFU vs. Chantecler.**

(d)

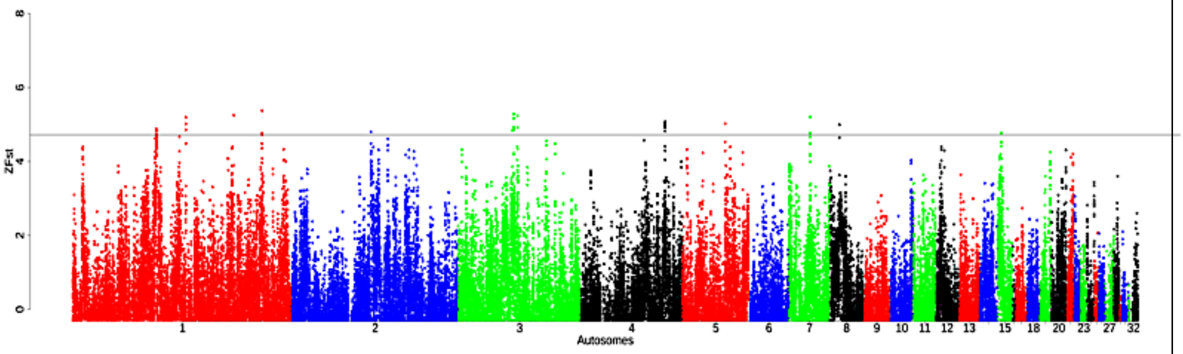

Manhattan plot of the distribution of *ZFST* values in **Omani vs. Chantecler.**

(e)

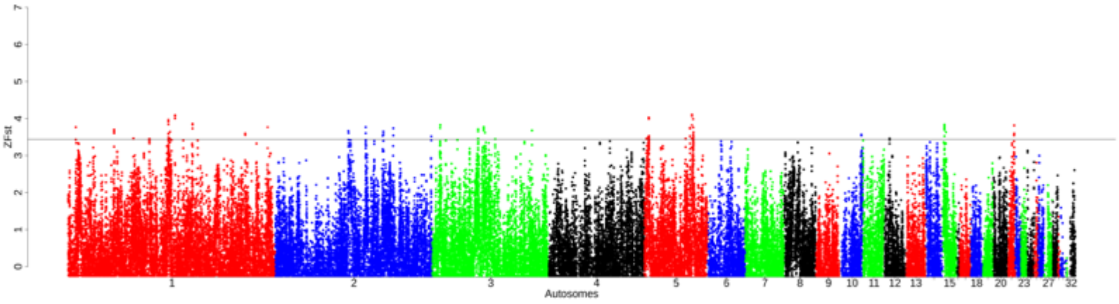

Manhattan plot of the distribution of ZF_{ST} values in Fayoumi **vs. Chantecler.**

(f)

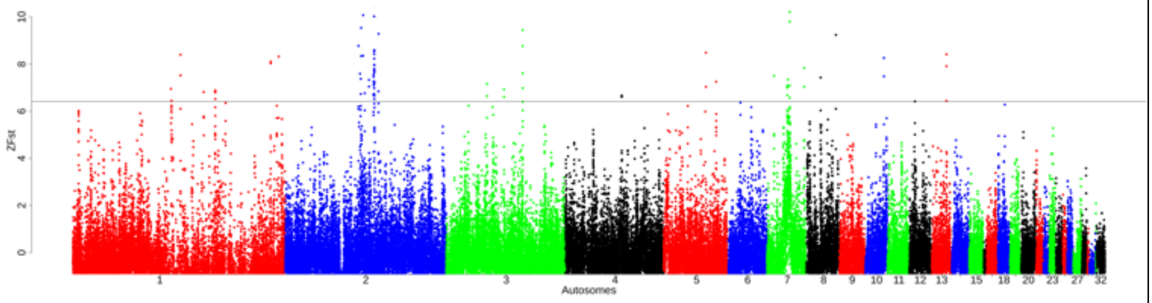

Manhattan plot of the distribution of ZF_{ST} values in **EPFCA vs EPFWA *.**

Fig S4 ZF_{ST} plots for comparisons. (a–e) Manhattan plots of standardized ZF_{ST} statistics for (Arabian Peninsula vs. **Chantecler** population), (Fayoumi vs. **Chantecler**), and Ethiopian populations (EPFCA vs EPFWA*) comparisons. The dashed horizontal lines represent the cut-off for extreme ZF_{ST} scores, corresponding to the upper 0.001 percentile of ZF_{ST} values.

(a)

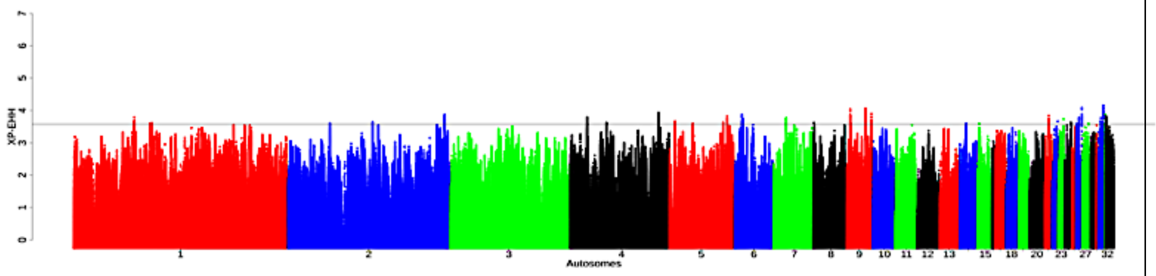

Manhattan plot of the distribution of *XP-EHH* values in **BL-KFU_vs_DT-China.** The horizontal dashed line indicates the threshold significant at *XP-EHH* =~ 3.62

(b)

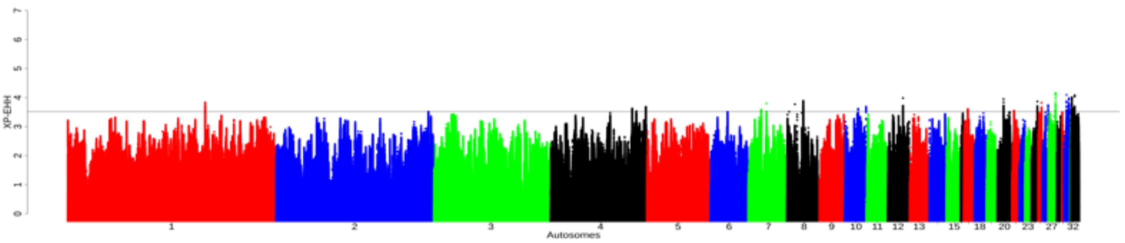

Manhattan plot of the distribution of *XP-EHH* values in **Br-KFU vs. DT-China.** The horizontal dashed line indicates the threshold significant at *XP-EHH* =~ 3.61

(c)

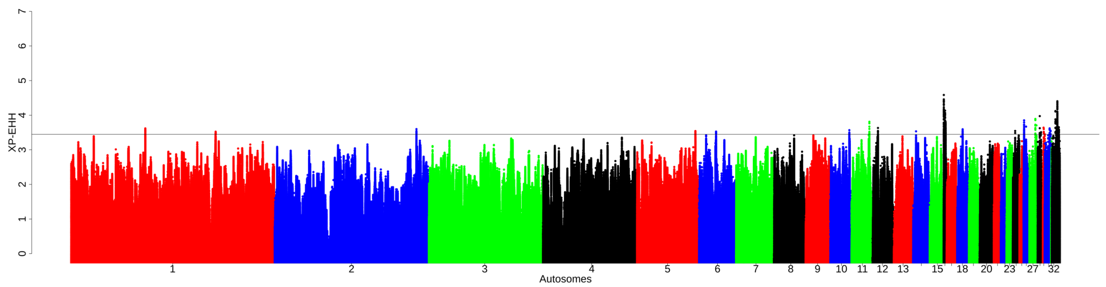

Manhattan plot of the distribution of ***XP-EHH*** values in **Omani vs DT-China.**

(d)

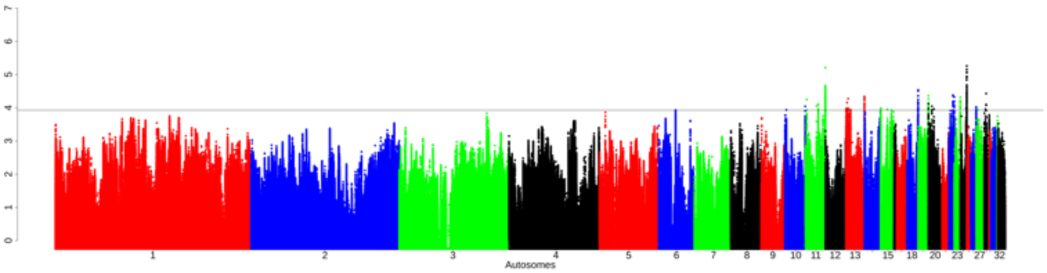

Manhattan plot of the distribution of values in **Bl-KFU vs. Chantecler.**

(e)

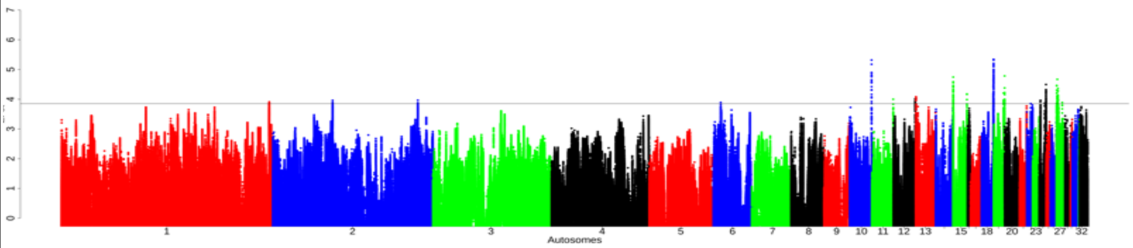

Manhattan plot of the distribution of *XP-EHH* values in **BU-VI vs. Chantecler.**

(f)

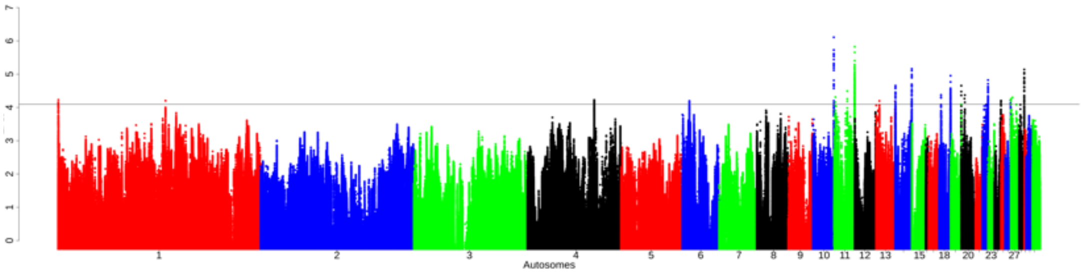

Manhattan plot of the distribution of ***XP-EHH*** values in **Br-KFU_vs_Chantecler.**

(g)

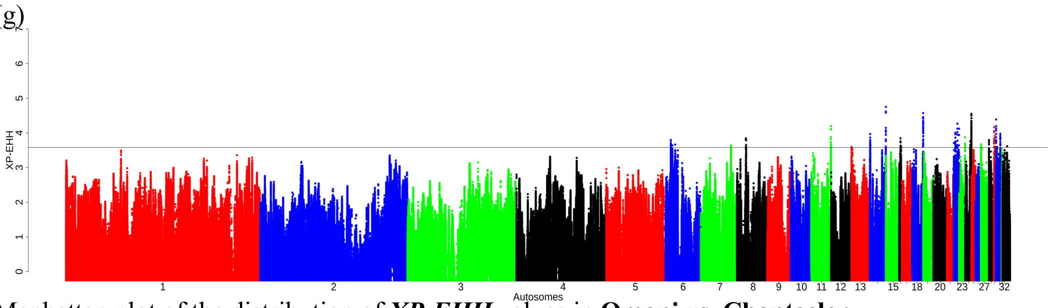

Manhattan plot of the distribution of ***XP-EHH*** values in **Omani vs. Chantecler.**

(h)

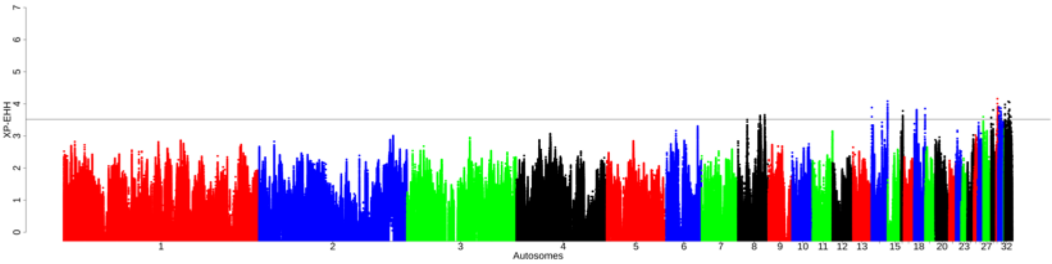

Manhattan plot of the distribution of ***XP-EHH*** values in **Fayoumi vs. DT-China.**

(i)

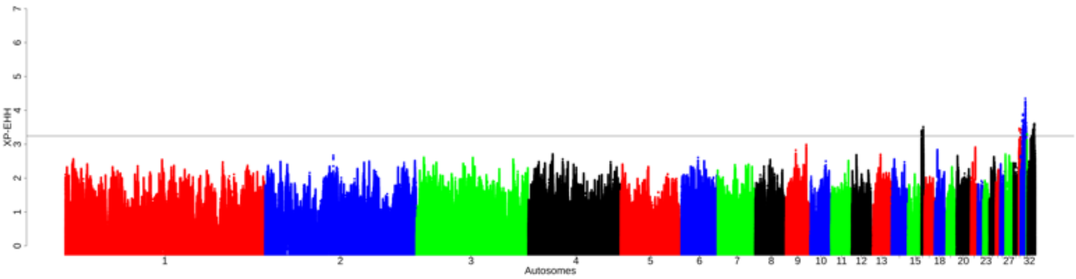

Manhattan plot of the distribution of ***XP-EHH*** values in **Fayoumi vs. Chantecler population.**

(j)

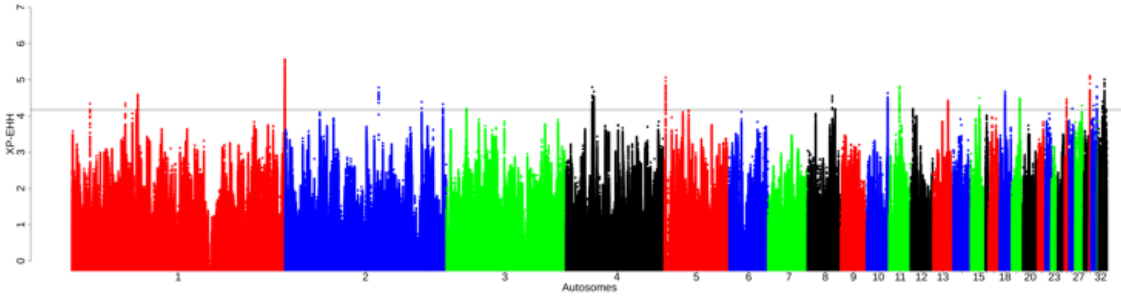

Manhattan plot of the distribution of *XP-EHH* values in **EPFCA vs EPFWA *.**

Fig S5 *XP-EHH* plots for comparisons. (a–g) Manhattan plots of standardized *XP-EHH* statistics for (Arabian Peninsula vs. DT-China), (Arabian Peninsula vs. Chantecler), (Fayoumi vs. Chantecler population), (Fayoumi vs. DT-China and Chantecler), and (EPFCA vs EPFWA**)** comparisons. The dashed horizontal lines represent the cut-off for extreme XP-EHH scores, corresponding to the upper 0.001 percentile of XP-EHH values.
